# The Colorectal Cancer Microbiota Alter Their Transcriptome To Adapt to the Acidity, Reactive Oxygen Species, and Metabolite Availability of Gut Microenvironments

**DOI:** 10.1128/msphere.00627-22

**Published:** 2023-02-27

**Authors:** Matthew T. F. Lamaudière, Ramesh Arasaradnam, Gareth D. Weedall, Igor Y. Morozov

**Affiliations:** a Centre for Sports, Exercise and Life Sciences, Coventry University, Coventry, United Kingdom; b Division of Biomedical Sciences, Warwick Medical School, University of Warwick, Warwick, United Kingdom; c Department of Gastroenterology, University Hospitals of Coventry and Warwickshire, National Health Service Trust, Coventry, United Kingdom; d University of Leicester, Leicester, United Kingdom; e School of Biological and Environmental Sciences, Liverpool John Moors University, Liverpool, United Kingdom; University of Michigan-Ann Arbor

**Keywords:** colorectal cancer, metatranscriptome, gut microbiota, reactive oxygen species, acidity, virulence

## Abstract

The gut microbiome is implicated in the pathology of colorectal cancer (CRC). However, the mechanisms by which the microbiota actively contribute to disease onset and progression remain elusive. In this pilot study, we sequenced fecal metatranscriptomes of 10 non-CRC and 10 CRC patient gut microbiomes and conducted differential gene expression analyses to assess any changed functionality in disease. We report that oxidative stress responses were the dominant activity across cohorts, an overlooked protective housekeeping role of the human gut microbiome. However, expression of hydrogen peroxide and nitric oxide-scavenging genes was diminished and augmented, respectively, positing that these regulated microbial responses have implications for CRC pathology. CRC microbes enhanced expression of genes for host colonization, biofilm formation, genetic exchange, virulence determinants, antibiotic, and acid resistances. Moreover, microbes promoted transcription of genes involved in metabolism of several beneficial metabolites, suggesting their contribution to patient metabolite deficiencies previously solely attributed to tumor cells. We showed *in vitro* that expression of genes involved in amino acid-dependent acid resistance mechanisms of meta-gut Escherichia coli responded differently to acid, salt, and oxidative pressures under aerobic conditions. These responses were mostly dictated by the host health status of origin of the microbiota, suggesting their exposure to fundamentally different gut conditions. These findings for the first time highlight mechanisms by which the gut microbiota can either protect against or drive colorectal cancer and provide insights into the cancerous gut environment that drives functional characteristics of the microbiome.

**IMPORTANCE** The human gut microbiota has the genetic potential to drive colorectal cancer onset and progression; however, the expression of this genetic potential during the disease has not been investigated. We found that microbial expression of genes that detoxify DNA-damaging reactive oxygen species, which drive colorectal cancer, is compromised in cancer. We observed a greater activation of expression of genes involved in virulence, host colonization, exchange of genetic material, metabolite utilization, defense against antibiotics, and environmental pressures. Culturing gut Escherichia coli of cancerous and noncancerous metamicrobiota revealed different regulatory responses of amino acid-dependent acid resistance mechanisms in a health-dependent manner under environmental acid, oxidative, and osmotic pressures. Here, for the first time, we demonstrate that the activity of microbial genomes is regulated by the health status of the gut *in vivo* and *in vitro* and provides new insights for shifts in microbial gene expression in colorectal cancer.

## INTRODUCTION

Colorectal cancer (CRC), the second most deadly cancer (1), lacks early diagnostic markers and develops over decades through accumulation of sporadic genetic lesions in >90% of cases (2). Metagenome-based analyses have shown that lifestyle and environmental factors are codeterminants of sporadic CRC (3, 4); however, how they promote neoplasia is mostly attributed to triggering chronic inflammation. Inflammation can be controlled by and effect the gut microbiome; hence, the microbiota and their metabolism appear to be the vital link in the development of sporadic CRC. The altered functional potential, namely, changes in gene abundance, of the gut microbiota in CRC is well known (5); however, the relevance to pathology of the host is not yet known. It has been recently found that the gene abundance and corresponding transcript levels are not always comparable (6); measuring variability in transcript levels minimizes the potential for misinterpretation of microbiome function in health and disease. Therefore, understanding how the CRC environment affects microbial patterns of gene expression will uncover the potential mechanisms by which the gut microbiota might directly influence long-term host epithelial health.

The wider colorectal environment and tumor microenvironment (TME) exhibit features such as inflammatory phenotypes, reactive oxygen species (ROS), and reactive nitrogen species (RNS), respectively (7), O_2_ and NO_3_^−^ saturation (8) and altered metabolite availability such as glucose, lactate, and iron (9). Coinciding with altered cancer cell metabolism, namely, the Warburg effect (glycolysis even in the presence of oxygen), a significantly acidic mucosa and intestinal lumen pH is reported in CRC and various other gut pathologies (10). The failure of cancerous epithelial cells to differentiate results in a lack of protective mucus production and compromised tight junction assembly; the resultant weakened barrier function (11) leaves the host susceptible to microbial colonization and inflammation. This coincides with biofilm formation in the CRC gut and the expansion of pathogenic species (12). Understanding the impact of the complex CRC gut environment on the specific activity of the microbiota is crucial to uncovering the mutualistic interplay between host and microbe in this primarily noncommunicable disease.

We characterized the microbial transcriptional profiles (metatranscriptomes) of fecal samples from 10 non-CRC and 10 CRC patient guts. We grouped differentially expressed genes and pathways with respect to their known function(s) and aligned them to known and new potentially influential phenotypes of the microbiome in CRC and homeostasis. Based on observed microbial responses, we also inferred specific environmental conditions, either global, transient, or spatial, which could have elicited these transcriptional shifts in disease. Finally, we investigated the transcriptional patterns of Lys- and Arg-dependent acid resistance mechanisms in CRC and control gut Escherichia coli in response to different environmental (acidic, osmotic, and oxidative) pressures *in vitro*.

## RESULTS

Through principal-component analysis (PCA), we established that “health condition,” namely CRC, had the primary effect on global transcription by the gut microbiome, patient metadata (e.g., age, sex, and body mass index [BMI]) had little to no influence over transcriptome composition (Fig. S1 and S2A). While age might look significantly variable between groups, averaging 35 years in controls and 71 years in cases, the PCA (Fig. S1B) shows some overlapping between the two groups (under and over 73 years, deemed elderly). The effect of CRC on microbial gene expression between the two cohorts exhibits distinct separate clustering (Fig. S2A and B; *P = *0.025 analysis of similarity [ANOSIM] of Bray-Curtis dissimilarity). Previous DNA-based analyses have suggested shifts in individual genus in the gut microbiome between young and elderly groups (13–16). However, a microbiome-wide effect of age-related shifts has not yet been shown. Other studies have described a core microbiota common to the 29 to 39 years age group, as well as the 39 to 49, 49 to 59, and 59 to 69 years age groups (17). Furthermore, longitudinal gut microbiotas of healthy adults have been shown to be relatively stable, even over decades (18). Therefore, while we cannot completely rule out the influence of age to our metatranscriptome data analysis, we infer that it is CRC that is the distinct determining factor for differences in microbial gene expression. Sequences were mapped to annotated gene sequences and assigned to curated subsystems of functional roles (SEED subsystems hierarchy level 3 in MetaGenomics-Rapid Annotation of microbial genomes using Subsytems Technology (MG-RAST)). The differential relative transcript level of these subsystems was compared between control and CRC samples to characterize the CRC-associated functional transcriptome. Of the 1,361 curated subsystems, 901 were identified in this analysis (Table 1 in Data Set S1). A total of 49 subsystems were significantly over-represented and 24 were significantly under-represented across all samples, with 261 genes of 6,495 differentially expressed, 182 upregulated, and 79 downregulated in CRC (Fig. S3A; Table 2 in Data Set S1). These differentially expressed subsystems and genes of the gut microbiota represent a CRC-specific transcriptional signature.

10.1128/msphere.00627-22.1 FIG S1 Patient metadata show limited to no effect on microbial gene expression during colorectal cancer. Scatter plots of variation across Dim1 and Dim2 of microbial transcriptome-wide gene expression. (A) Gender. Microbial gene expression was not affected by participant sex. (B) Age (above and below 73 years, elderly and nonelderly). Microbial gene expression shows clustering in the over 73 group, while overlapping with the under 73 group. (C) Smoking status. Microbial gene expression was not affected by participant smoking status. (D) Body mass index (BMI) (obese, >30 and nonobese, <30). Microbial gene expression was not affected by participant obesity status. (E) Carcinoembryonic antigen (CEA) range <6 μg/L. Microbial gene expression was not affected by patient CEA range (grouped above and below 4 μg/L). (F) Cancer stage (early stage, T2 to T3; late stage, T4). Cancer stage may affect microbial gene expression, colorectal cancer (CRC) patient groups showed weak clustering. NA, not applicable. Download FIG S1, TIF file, 2.6 MB. Copyright © 2023 Lamaudière et al. 2023 Lamaudière et al. https://creativecommons.org/licenses/by/4.0/ This content is distributed under the terms of the Creative Commons Attribution 4.0 International license.

10.1128/msphere.00627-22.2 FIG S2 Colorectal cancer influences the microbial transcriptome. (A) Principal-coordinate analysis (PCoA) scatter plot of Bray-Curtis dissimilarity (β-diversity) across Dim1 and Dim2 of microbial transcriptome-wide gene expression. Blue circles represent control samples, and red triangles represent CRC samples. The samples demonstrated clustering with respect to origin as CRC influences variation in microbial gene expression. (B) Analysis of similarity (ANOSIM) of Bray-Curtis dissimilarity revealed that intrasample diversity is significantly lower than intersample dissimilarity between CRC and control. (C) The α-diversities across all samples as assessed by the Gini-Simpson index. Download FIG S2, TIF file, 0.9 MB. Copyright © 2023 Lamaudière et al. 2023 Lamaudière et al. https://creativecommons.org/licenses/by/4.0/ This content is distributed under the terms of the Creative Commons Attribution 4.0 International license.

10.1128/msphere.00627-22.3 FIG S3 (A) Microbial transcriptome-wide changes in gene expression during colorectal cancer compared to controls. The volcano plot of −Log10 Benjamini-Hochberg-adjusted (false discovery rate [FDR] <0.1) *P* values for transcriptome-wide gene expression against their respective Log2 fold changes. Blue and red dots represent significantly down- and upregulated genes between CRC and control groups, respectively. Gray dots represent genes with no changes in expression. Of 6,495 genes, 261 (4,856 shown) have significantly altered expression during CRC. (B) CRC microbiota depletes the host of beneficial metabolites and micronutrients. The CRC-associated microbiota transcribe *n*-butyrate synthesizing acetyl-CoA:acetoacetyl-CoA transferase more readily but 3-hydroxybutyryl-CoA dehydratase and 3-ketoacyl-CoA thiolase to a lesser extent. Betaine aldehyde dehydrogenase, glycine betaine specific transporter OpuD, and the precursor for nonspecific OmpC, which are preferentially expressed at low pH and in high osmolarity, were overexpressed alongside the subsystem for carnitine metabolism. Upregulated transcription of CaiF, CaiA, and OpuCA (the osmotically activated l-carnitine/choline ABC transporter) are evidence of higher carnitine metabolism rates by the microbiome. The biosynthesis and uptake of glycine betaine are also more pronounced in CRC. Enterobacteriales upregulated expression of the osmoprotectant ABC transporter YehZYXW (Fig. 3A), the pleotropic role of which is important for the accumulation of the glycine betaine osmolyte under hyperosmotic stress, nutrient starvation, and acidic environments (1). The bacterial production of the sphingolipid, ceramide, and the galactocerebrosidase precursor protein in CRC were downregulated. This may lead to a reduced supply of ceramide to the host, hence attenuating apoptosis, prolonging cell survival, and enhancing tumor growth and glucose metabolism. Transcription of the Cbr modules of the predicted cobalamin energy-coupling factor (ECF) transporter was more active in cancer along with expression of the CrdX gene, a cobalamine-related hypothetical metal-binding protein. Expression of genes that respond to purine availability to repress purine biosynthesis was enhanced in CRC. Levels of hypoxanthine oxidase XdhD, 5′-nucleotidase, and 2′,3′-cyclic-nucleotide 2′-phosphodiesterase transcription, which produce adenosines, were diminished. The genes implicated in regulating microbial vitamin B_2_ (Fig. 2B and C), B_6_, and B_12_ and their derivatives were differentially expressed. Three predicted pyridoxine and cobalamin ECF transporters and cobalamin metal-binding protein CrdX were expressed to a higher degree during CRC. Black diamonds, *n*-butyrate and ceramide; pink diamonds, carnitine, serotonin and Se; green diamonds, purines; purple diamonds, vitamins. Download FIG S3, TIF file, 2.2 MB. Copyright © 2023 Lamaudière et al. 2023 Lamaudière et al. https://creativecommons.org/licenses/by/4.0/ This content is distributed under the terms of the Creative Commons Attribution 4.0 International license.

10.1128/msphere.00627-22.8 DATA SET S1 Sequences mapped against subsystems of functional roles (SEED subsystems hierarchy level 3 in MG-RAST), sequences mapped against specific genes of functional roles (SEED subsystems hierarchy level 4 in MG-RAST), and compositional analysis (16S rRNA gene-based abundance) of the aerobic cultures. Download Data Set S1, XLSX file, 0.9 MB. Copyright © 2023 Lamaudière et al. 2023 Lamaudière et al. https://creativecommons.org/licenses/by/4.0/ This content is distributed under the terms of the Creative Commons Attribution 4.0 International license.

### Oxidative stress responses are housekeeping functions of the microbiome irrespective of gut health status.

**FIG 1:**
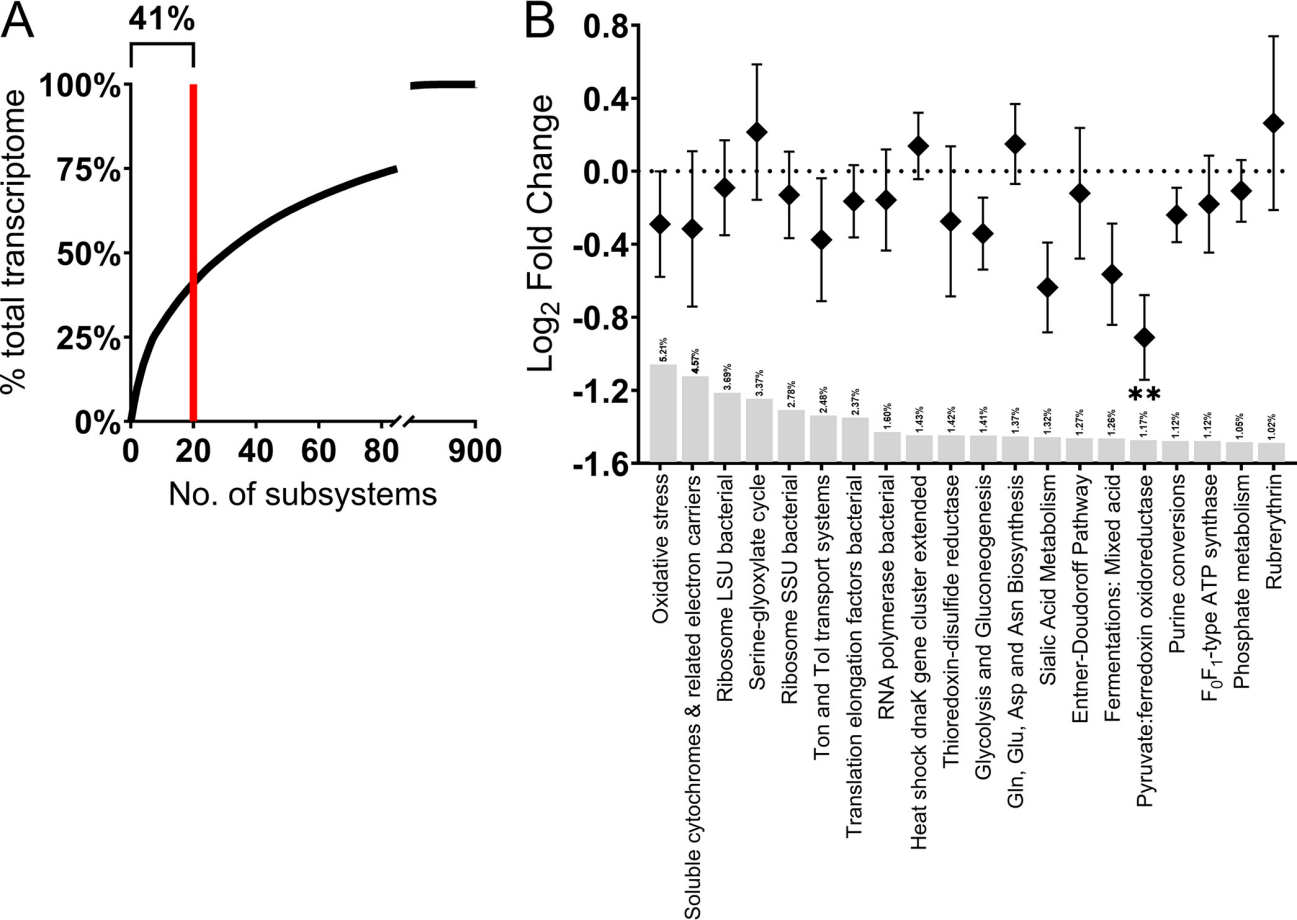
The core transcriptome of the gut microbiota is mostly maintained in colorectal cancer. (A) Threshold of subsystems considered core, 20 subsystems of 900 identified contribute 41% of total transcriptome activity. Asterisks denote statistically significant differences between the health and CRC cohorts. **, *P*_adj_ < 0.008. (B) Metatranscriptional profile of the most prevalently expressed, “core” subsystems across all samples in both colorectal cancer (CRC) and non-CRC cohorts. The gut microbiota generate biomass primarily through glycolysis-gluconeogenesis, the serine-glyoxylate cycle, purine metabolism, amino acids (Gln/Glu and Asn/Asp) biosynthesis and ions, vitamins, and iron transport. Microbial metabolism of sialic acid, a terminal modification of host colonocytes and mucus, also appears to be a common housekeeping activity of the human gut microbiome. We also observed that oxidative stress responses (Ton and Tol transport systems, thioredoxin reduction, heat shock *dnaK* gene cluster subsystems) featured within the core transcriptome of both healthy and CRC-associated microbiota. The individual subsystem contribution to the overall transcriptome is displayed as a percentage above gray bars.

The housekeeping activity of the human gut microbiome has been studied at the genomic and transcriptomic levels in healthy adults (19); however, it is yet to be elucidated in CRC. The most active subsystems, the core transcriptome (each constituting >1% of the total transcriptome) accounted for ~40% of total microbial activity (Fig. 1A); only one, pyruvate:ferrodoxin oxidoreductase that decarboxylates pyruvate to acetyl-CoA in anaerobes, showed a significant reduction in CRC (Fig. 1B). This “core” transcriptome appears to be responsible for housekeeping activities, biosynthesis, and energy production. Interestingly, oxidative stress responses dominate, despite inflammation/oxidative stress being long considered a disease-specific phenotype. This indicates that the microbiome plays a key role in mediating the level of ROS within the gut.

### Gut microbiota alters the level of enzymatic and nonenzymatic antioxidative activities in CRC.

**FIG 2:**
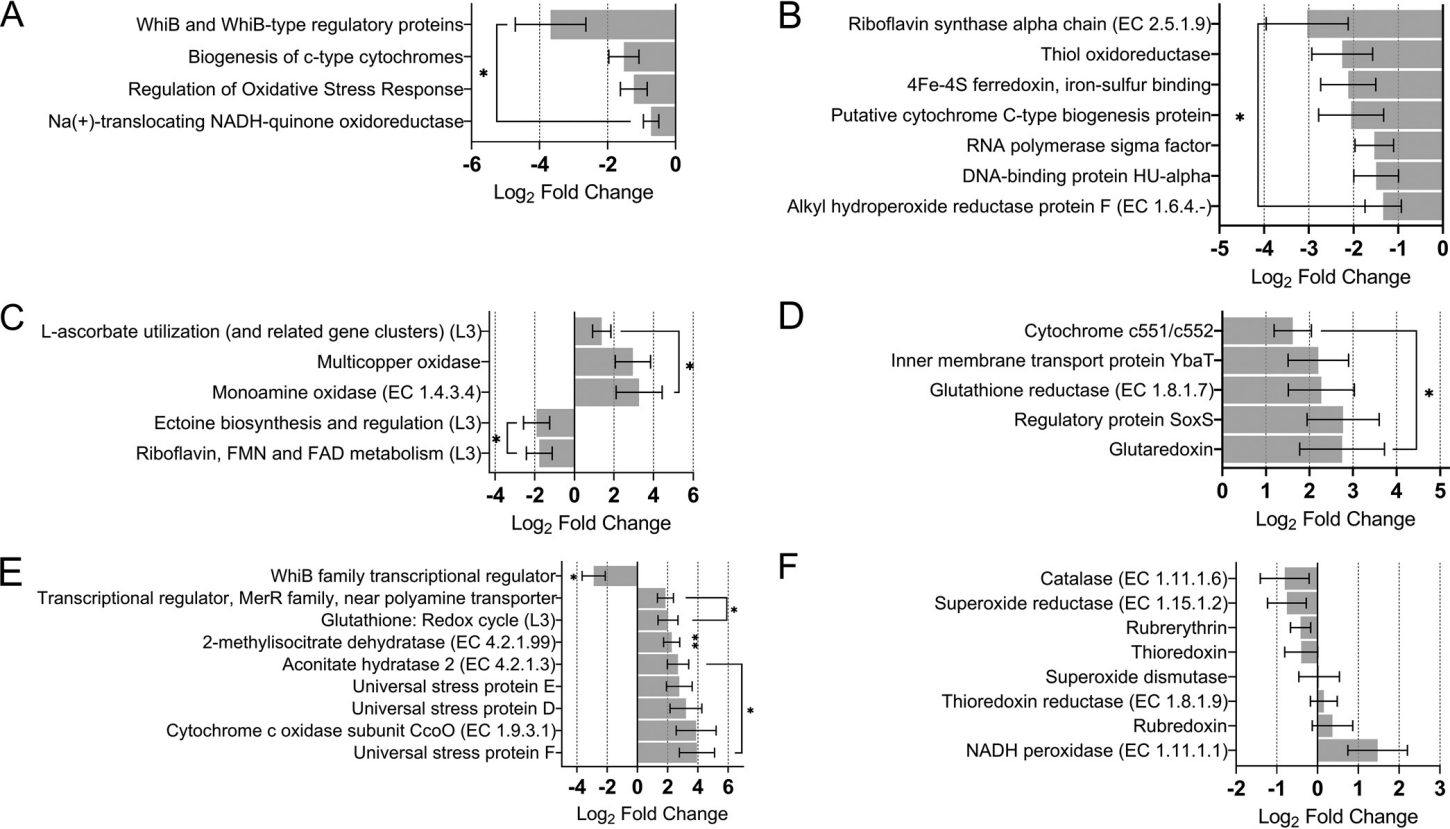
The microbiome response to H_2_O_2_ is diminished, and the response to NO is increased in colorectal cancer despite high background levels of oxidative stress activities in health and disease. (A) Activity of subsystems involved in modulation of oxidant levels are repressed in CRC. These subsystems involve sensors of oxidative stress (87), reduction of quinones (88), and *c*-type cytochrome and the antioxidant riboflavin (vitamin B_2_) synthesis (89). (B) Expression of specific genes related to oxidative damage in CRC. The expression of RNA polymerase sigma factor, a universal regulator of microbial oxidative stress response, the DNA-binding protein HU-α, a bacterial histone-like protein which displays high affinity to damaged DNA and plays a part in the oxidative DNA damage response (90), was also significantly downregulated. The expression of 4Fe-4S ferredoxin, thiol oxidoreductase and putative *cytochrome c*-type biogenesis protein genes, prominent regulators of redox status and global nitrogen and sulfur cycles, was also significantly diminished. Transcription of the riboflavin synthase and alkyl hydroperoxide reductase genes was also downregulated. (C) CRC gut microbiota express genes for the utilization and oxidation of several nonenzymatic antioxidants such as ectoine and l-ascorbate. (D) Microbiota in CRC maintains a reduced gut environment. Expression of cytochrome *c*_551_/*c*_552_ and regulatory protein SoxS, a superoxide response regulon transcriptional regulator (91), was upregulated. The CRC microbiota showed a high uptake of Se (selenate and selenite), an essential element that is critical for production and activity of antioxidative selenoproteins. Selenoproteins are vital for host immunity and antiviral defense, which enhanced levels of the inner membrane transport protein YbaT, and selenoproteins O synthesis have been observed (92), correlating with higher Se uptake. (E) The CRC gut contains elevated O_2_^−^ and NO levels, and the expression of genes the activity of which is implicated in their removal was elevated. Transcription of cytochrome *c* oxidase, CcoO subunit, with high NO reductase activity and *MerR*, a transcriptional factor that regulates NO defense (93), was significantly overactive in CRC. Synthesis of NO-induced universal stress proteins D, E, and F (94) was significantly enhanced. Aconitate hydratase 2 and 2-methylisocitrate dehydratase, the expression of which is negatively regulated by NO, are also transcribed to a higher degree. (F) A high level of reactive oxygen species (ROS)-reducing activity appears to be a housekeeping characteristic of the gut microbiome. Expression of major ROS-reducing genes was maintained in a health status-independent manner. (L3) denotes a subsystem. *, *P ≤ *0.05; **, *P ≤ *0.01. FAD, flavin adenine dinucleotide; FMN, flavin mononucleotide.

The majority of CRC cases have a sporadic origin and result from gradual accumulation of somatic mutations in glandular epithelial cell DNA (20). This is attributed to the deleterious effects of ROS and RNS on DNA integrity and repair. We found that microbial ROS/RNS-scavenging activities were altered in CRC. Unexpectedly, several ROS-reducing subsystems were significantly repressed in CRC (Fig. 2A). Alkyl hydroperoxide reductase and thiol oxidoreductase scavenge H_2_O_2_, the most potent DNA-damaging agent (noncharged H_2_O_2_ is easily taken up by colonocytes). Transcription of nine genes alongside genes involved in oxidative DNA damage responses was significantly downregulated (Fig. 2B).

Analysis via HUMANn3 showed a limited set of specific bacteria are responsible for expression of ROS-reducing genes in the human gut in CRC (Table 1). Interestingly but unsurprisingly, Bacteroides spp. appear to be the dominant genus in expression of these ROS detoxification factors, while also being the most active genus in the community.

**TABLE 1:** Species-specific expression of genes involved in ROS reduction in CRC^*a*^

Gene	Proposed mechanism	Organisms
Regulatory protein SoxS	Transcriptional activator of the superoxide response regulon	Parabacteroides distasonis Fusicatenibacter saccharivorans
Glutaredoxin	Reduce disulfide bonds or catalyse reversible protein glutathionylation or deglutathionylation	Bacteroides vulgatus Bacteroides dorei Bacteroides plebeius
Alkyl hydroperoxide reductase	Reduce hydrogen peroxide and organic hydroperoxides	Bacteroides uniformis Catenibacterium mitsuokai Bacteroides vulgatus Flavonifractor plautii Bacteroides dorei Bacteroides stercoris
4Fe-4S ferredoxin	Intracellular electron carrier with low values of reduction potential	Bacteroides dorei Bacteroides vulgatus Bacteroides uniformis Bacteroides sartorii Collinsella aerofaciens
Na^+^-translocating NADH-quinone reductase subunits A to F	A respiratory enzyme (complex I) that catalyzes the electron transfer from NADH to quinone in the cytoplasmic membrane	Bacteroides vulgatus Parabacteroides sp. HGS0025 Bacteroides dorei Bacteroides massiliensis Parabacteroides distasonis Bacteroides caccae Parabacteroides merdae Bacteroides ovatus Bacteroides thetaiotaomicron Bacteroides sp. D2 Bacteroides xylanisolvens Catenibacterium mitsuokai Bacteroides fluxus Bacteroides stercoris Ruminococcus torques Bacteroides clarus Bacteroides cellulosilyticus
Superoxide dismutase	Metalloenzymes that catalyze the dismutation of superoxide anion, superoxide into molecular oxygen, and hydrogen peroxide	Bacteroides dorei Bacteroides vulgatus Parabacteroides distasonis Bacteroides sartorii Intestinimonas butyriciproducens
Thiol peroxidase	Reduce hydrogen peroxide and lipid hydroperoxides to water and alcohols, respectively	Parabacteroides johnsonii Parabacteroides merdae Clostridium disporicum Bacteroides uniformis Bacteroides vulgatus Catenibacterium mitsuokai Bacteroides dorei Bacteroides thetaiotaomicron Bacteroides ovatus Bacteroides caccae Bacteroides fragilis Bacteroides sp. D2 Bacteroides xylanisolvens Bacteroides caecimuris Bacteroides cellulosilyticus
MerR family transcriptional regulator	Group of transcriptional factors that mediate (among numerous other functions) the oxidative stress response	Bacteroides uniformis Catenibacterium mitsuokai Bacteroides caccae Bacteroides thetaiotaomicron

a The table shows HUMAnN3 functional profiling of microbial reactive oxygen species (ROS)-reducing genes that exhibited significant regulation (except for superoxide dismutase) in colorectal cancer (CRC).

Bacteria can also produce and utilize protective nonenzymatic antioxidants. We found that the ectoine biosynthesis and regulation subsystem, which scavenges hydroxyl radicals and has anti-inflammatory activities (21), was downregulated in CRC (Fig. 2C). The l-ascorbate utilization subsystem displayed the opposite pattern of activity, suggesting l-ascorbate depletion. We observed upregulated transcription of the multicopper oxidase gene, involved in oxidation of different antioxidants, such as polyphenols, l-ascorbate, aromatic polyamines, and metal ions. Expression of the monoamine oxidase gene, the product of which is required for oxidative deamination of monoamines such as serotonin, a neurotransmitter present in the gastrointestinal mucosa (22), was also increased. This suggests that the gut microbiota can deplete secondary antioxidants during the cancer.

Higher levels of glutaredoxin and glutathione reductase expression in CRC demonstrates the significant role the microbiota plays in maintaining the redox status of the cell (Fig. 2D). Additionally, expression of several reactive species scavenging genes was significantly upregulated, suggesting that the CRC gut is featured with elevated O_2_^−^ and NO levels. Consistent with NO being a major RNS in CRC, primarily produced by neutrophils, expression of genes encoding the glutathione redox cycle pathway, which senses NO levels and some universal stress proteins (23), was increased (Fig. 2E). In the CRC gut, it would appear that NO and O_2_^−^ are the primary radicals to which the microbiota respond to, to different extents.

Unexpectedly, expression of genes involved in multiple ROS reduction pathways showed equally high levels of expression in both groups (Fig. 2F). Overall, these data showed that the microbial responses to O_2_^–^ were largely unchanged, those to H_2_O_2_ were lessened, and those to NO were enhanced during CRC. This strongly implies that the microbiome differentially responds depending on the nature of the ROS/RNS as a result of the gut health status. While a high level of background ROS reduction appears to be a housekeeping feature of the gut microbiome, fluctuations in compound-specific responses may mediate potential damaging effects.

### CRC-associated microbiota deplete the host of beneficial metabolites and respond to the acidic gut environment.

**FIG 3:**
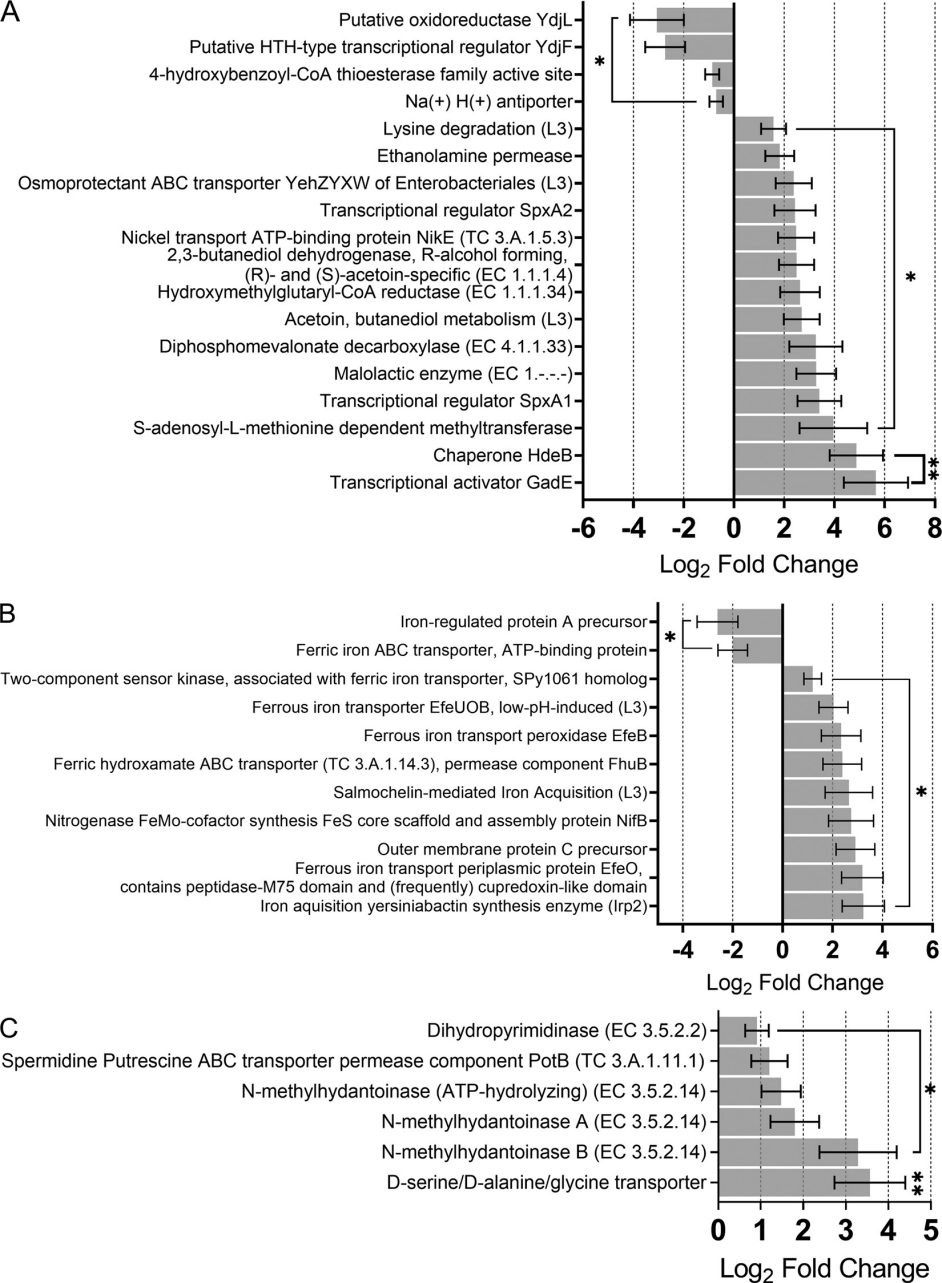
The CRC microbiome are adapted to the high acidity of the gut and metabolize host-required metabolites more readily. (A) Activity of glutamate-dependent acid resistance mechanisms through transcriptional activator GadE, glutamate transport membrane-spanning protein, and inner membrane transport protein YbaT (Fig. 2D), were all enhanced in CRC alongside the acid stress chaperone HdeB. Basic compounds such as ammonia (NH_3_^+^) can be produced by bacteria to offset low cellular pH, particularly from urea (95); the higher transcription of nickel transport ATP-binding protein NikE observed may be critical in providing the nickel for the activity of ureases that catalyze this conversion. Production of l-malate via expression of malate synthase and its conversion to L-lactate and CO_2_ by malolactic enzyme were also prominent features of the CRC microbiome, the activity of which is triggered at a pH of <2.3. Levels of ethanolamine permease transcription and acid stress-induced transcriptional regulators SpxA1 and SpxA2, which are virulence determinants in pathogens, were over-represented. Conversely, alkali pH-induced genes 4-hydroxybenzoyl-CoA thioesterase and putative helix-turn-helix (HTH)-type transcriptional regulator *YdjF* and *YdjL* oxidoreductase exhibited lower expression during cancer. (B) Iron uptake and transport-related genes are upregulated by the gut microbiota in CRC. Expression of *EfeO* and *EfeB*, iron acquisition yersiniabactin synthesis enzyme, outer membrane protein C precursor, ferric hydroxamate ABC transporter (a chelating mechanism of ferric iron [Fe^3+^] uptake), and two-component sensor kinase SPy1061 homolog that respond to iron availability and acid stress was more active. (C) The CRC gut microbiota actively metabolize exogenous DNA. Transcription of dihydropyrimidinase, *N*-methylhydantoinases A and B, guanine-hypoxanthine permease, d-serine/d-alanine/glycine transporter, phage-associated cell wall hydrolase, and *PotB* genes was increased in CRC. *, *P ≤ *0.05; **, *P ≤ *0.01.

**FIG 4:**
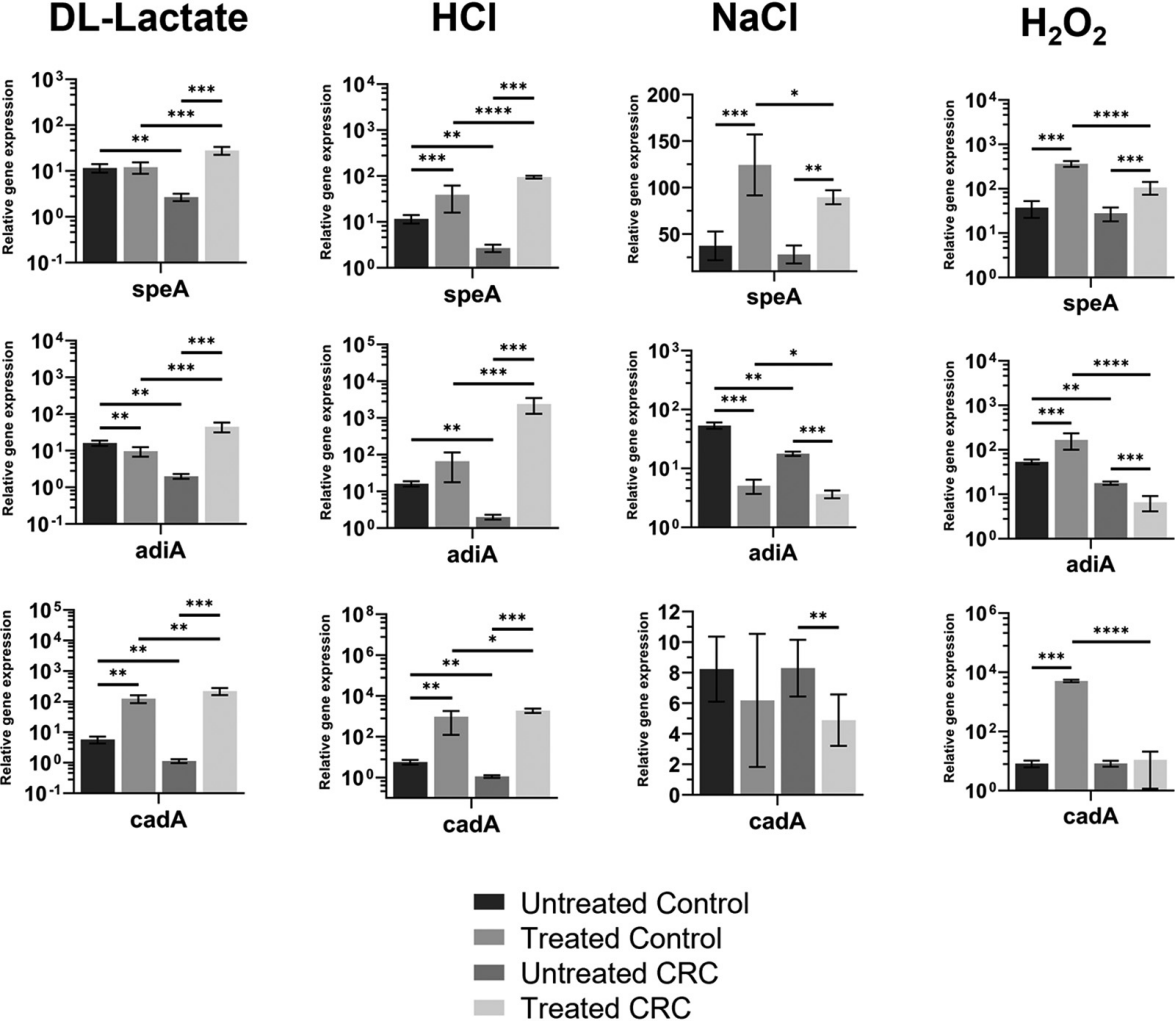
Amino acid Arg- and Lys-dependent acid defense mechanisms in E. coli are regulated by both environmental factors and the health status origin of bacteria. The level of expression of E. coli *speA* (Arg-decarboxylase), *adiA* (biosynthetic Arg-decarboxylase), and *cadA* (Lys-decarboxylase) genes was quantified by quantitative reverse transcription (qRT)-PCR in response to pH 3.5 adjusted by either dl-lactate or HCl, osmotic (5% NaCl), and oxidative (1.5 mM H_2_O_2_) pressures. Microbiota derived from CRC (untreated CRC and treated CRC) and control (untreated control and treated control) were aerobically cultured. Error bars denote standard deviation (treated, *n* = 9; untreated, *n* = 6). Asterisks represent statistically significance. *, *P ≤ *0.05; **, *P ≤ *0.01; ***, *P ≤ *0.001; ****, *P ≤ *0.0001.

It has long been known that the pH of the colorectum can drop to levels as low as 2.3 to 3.4 during severe disease (24). However, the impact on microbial physiology remained unknown. We observed evidence of microbial adaptation to highly acidic conditions during CRC, at the molecular (Fig. 3A) and phylogenetic (25) levels. The Na^+^-H^+^ antiporter subsystem, which modulates H^+^ potential across the bacterial membrane, was downregulated, implying high extracellular proton concentrations and low pH. A gamut of 19 differentially expressed genes support this assertion. We also observed that bacteria and archaea attempt to protect their cell membrane against H^+^ permeability. They may reinforce it with more cyclopropane fatty acids, overexpressing *S*-adenosyl-l-methionine-dependent methyltransferase (SAM MTase) (Fig. 3A) and unsaturated fatty acids through 3-hydroxydecanoyl-[acyl-carrier-protein] dehydratase (Fig. 4B; Text S1).

10.1128/msphere.00627-22.6 TEXT S1 Supplementary results and discussion. Download Text S1, DOCX file, 0.1 MB. Copyright © 2023 Lamaudière et al. 2023 Lamaudière et al. https://creativecommons.org/licenses/by/4.0/ This content is distributed under the terms of the Creative Commons Attribution 4.0 International license.

Iron availability and uptake has been associated with bacterial pathogenicity and is often linked to low environmental pH. Expression of the ferrous iron (Fe^2+^) transporter EfeUOB operon, which allows uptake of the relatively soluble Fe^2+^, was elevated by the cancerous gut microbiota (Fig. 3B). Functional profiling of metatranscriptomes in CRC via HUMAnN3 showed that *nifB* was expressed solely by Ruminococcus torques. The HUMAnN3 software did not identify species that expressed the *EfeUOB* operon. Instead, we mapped all 20 functionally annotated metatranscriptomes against the RefSeq “taxonomic expansion” database to establish contributing taxa. Through this approach, we identified that E. coli, Klebsiella pneumoniae, and Streptococcus spp. were responsible for expression of the *EfeUOB* operon in the CRC gut alongside *Enterobacteriaceae* and Citrobacter spp. However, the community downregulates its prominent nonchelating ferric iron uptake mechanism, ferric iron ABC transporter protein. Despite iron uptake being conducted by a core member of the housekeeping transcriptome (Ton-Tol transport systems) certain iron acquisition mechanisms are overexpressed in CRC, suggesting greater access to Fe^2+^.

The gut microbiota supply a range of health-maintaining essential metabolites to the host. For example, carnitine delivers fatty acids for β-oxidation to the mitochondria for energy production. Increased transcription of microbial genes involved in carnitine and Se uptake and catabolism was observed in CRC (Fig. S4). Moreover, the pattern of expression of *n*-butyrate-synthesizing genes indicates a switch in substrate specificity to favor acetone production from acetoacetyl-CoA (Text S1), suggesting a limited supply for the host to metabolize. We have shown the attenuated activity of 22 *n*-butyrate-producing species in the CRC microbiome (25), which corroborates these data. Carnitine is important for osmotic adaptation of the microbiota, suggesting that they are under increased osmotic stress. Microbial uptake of queuosine (Q), a precursor base of modified Q_34_-tRNA in bacteria and eukaryotes, critical for translation fidelity, a contributor to human health (26), was elevated in CRC. The microbiota also reduced transcription of genes implicated in ceramide production, an apoptosis activator, and enhanced vitamin B_2_, B_6_, and B_12_ uptake gene expression, further suggesting depletion of the host for essential compounds.

10.1128/msphere.00627-22.4 FIG S4 Microbial metabolic activity switches from carbohydrates in health to amino acids, aromatic compounds, and nucleic acids in CRC. (A) Anaerobic oxidative degradation of l-ornithine is upregulated in CRC alongside degradation of branched-chain amino acids (Iso, Val, and Leu). Microbial expression of l-lysine catabolism, phenylacetyl-CoA catabolic pathway, auxin biosynthesis, glutathione: biosynthesis and γ-glutamyl cycle subsystems was augmented. Transcription of glutamate transport membrane-spanning protein and genes involved in amino acid degradation, 2-oxoglutarate dehydrogenase, arginine deiminase, and threonine catabolic operon transcriptional activator TdcA, *S*-adenosyl-l-methionine-dependent methyltransferase was enhanced in cancer. PaaE ketothiolase (involved in phenylacetic acid degradation in aerobic conditions) was also expressed to a greater extent. (B) Expression of 30 genes that are involved in metabolism of carbohydrates was significantly attenuated in cancer, while expression of fructose metabolizing and transporting genes was elevated. (C) Methanogenic and biosynthetic metabolic features of the CRC microbiome coincide with local starvation. Archaeal growth and methanogenesis related gene expression in the CRC gut was enhanced. Coenzymes M, B, and F420 synthesis displayed amplified transcription along with methylotrophic pathway components methanol:corrinoid methyltransferase and methanol methyltransferase corrinoid protein genes, all crucial components in CH_4_ production by methanogenic microorganisms. Fibrillarin, Nop5/Nop56, translation initiation factor 2β, glycerol-1-phosphate dehydrogenase [NAD(P)], hydroxymethylglutaryl-CoA reductase, and archaeal riboflavin synthase genes that synthesize cell wall components all displayed increased expression in the cancerous gut. (D) Pyruvate metabolism-associated gene expression was repressed through weakened microbial transcription of the pyruvate:ferredoxin subsystem (Fig. 1B) and pyruvate-flavodoxin oxidoreductase contained therein, required for aerobic acetyl-CoA production. Transcription of biosynthetic glyoxylate bypass genes, circumventing the decarboxylating steps of the tricarboxylate cycle, and the whole subsystem itself was upregulated, including aconitate hydratase 2 (Fig. 2E), isocitrate lyase, and malate synthases. Succinate dehydrogenase subunits of respiratory complex II that supply electrons to the respiratory chain, 2-oxoglutarate dehydrogenase, and LamB all responsible for succinate metabolism were upregulated by the microbiome, in conjunction with downregulation of the transcriptional repressor of the myo-inositol catabolic operon, which can also be utilized in succinate synthesis. Genes encoding complex III-related (cytochrome *o* ubiquinol oxidase, subunits I and III), IV-related (cytochrome *c* oxidase and heme O synthase), and V-related (V-type ATP synthase, subunits C, E, and I) components to reduce oxygen to H_2_O were upregulated. Complex I, Na^+^-translocating NADH-quinone reductase subunit E and the FMN-producing pathway were less active in CRC. Gene expression in response to nutrient starvation, e.g., amino acid, such as ribosome hibernation protein YfiA was a feature of the CRC microbiome. The microbiota also enhanced expression of the YeaH and YeaG, which are implicated in nitrogen starvation responses. The level of transcripts for aromatic hydrocarbon utilization transcriptional regulator CatR, hypothetical nudix hydrolase YeaB, La protease and glutamate synthase, α-subunit were increased in malignancy. Black diamonds, pyruvate; pink diamonds, trichloroacetic acid (TCA)/glyoxylate; green diamonds, TCA/succinate; purple diamonds, electron transport chain (ETC); lilac diamonds, starvation. Download FIG S4, TIF file, 2.2 MB. Copyright © 2023 Lamaudière et al. 2023 Lamaudière et al. https://creativecommons.org/licenses/by/4.0/ This content is distributed under the terms of the Creative Commons Attribution 4.0 International license.

The CRC-associated microbiota activated expression of hydantoin uptake and metabolism genes, the products of oxidation of cytosine and thymine bases of dead cell DNA (27) (Fig. 3C). Lysis of bacterial cells due to higher bacteriophages activity and biofilm formation (28) in CRC may in part explain the availability of exogenous hydantoins and purines. Cell-free (cf) DNA in the gut may also be available from the accelerated death of tumor and immune cells (29). Additionally, transport of spermidine and putrescine (biogenic amines, products of fatty and amino acid breakdown from decaying cells/tissues) was also significantly increased. We observed that xanthine/xanthosine-, inosine-, and guanine-metabolizing genes were upregulated by the microbiome, a source of microbial ROS, while adenine/adenosine salvage genes were downregulated (Fig. S4; Text S1). We found that expression of a number of genes involved in carbohydrate metabolism was diminished in CRC with the opposite activities for utilization of amino acid and aromatic compounds (Text S1; Fig. S5A and B). This suggests a switch from carbohydrates in health to amino acids and aromatic compounds metabolism in CRC. Archaeal methanogenesis activities and expression of microbial genes for biosynthetic pathways were enhanced in CRC (Text S1; Fig. S4C).

10.1128/msphere.00627-22.5 FIG S5 (A) The CRC microbiota exhibit an enhanced rate of growth. Genes required for global processes such as translation, replication, cell division, membrane, and cell wall biosynthesis displayed enhanced transcript levels under CRC conditions. Anaerobic photosynthetic growth may be diminished as photoporphyrin IX Mg-chelatase subunit H expression was downregulated, a possible reflection of less active Cyanobacteria (e.g., Xenococcus spp.) (2) in the CRC gut. (B) Microbial sporulation is diminished, while germination is dynamic in the CRC gut niche. Expression of WhiB-like transcription regulator (Fig. 2E) (necessary for sporulation in *Actinomycetes*), spore cortex-lytic enzyme precursor (required for completion of germination), and spore coat protein S (a major spore coat protein produced early in sporulation) are repressed, while transcription of the Gpr gene, which encodes a spore protease required for early-stage germination of spores, was increased in CRC. Download FIG S5, TIF file, 1.4 MB. Copyright © 2023 Lamaudière et al. 2023 Lamaudière et al. https://creativecommons.org/licenses/by/4.0/ This content is distributed under the terms of the Creative Commons Attribution 4.0 International license.

### Amino acid-dependent acid resistance mechanisms of E. coli derived from CRC and healthy guts are regulated differently.

Microbial RNA sequencing (RNA-seq) data argue that the CRC gut environment is more acidic, fluctuates in osmotic potential, and is less saturated with H_2_O_2_ compared to the control gut. The aerobic microbial populations of both conditions, grown in LB over 24 h until stationary phase, were enriched with 60% to 70% of E. coli based on 16S rRNA gene sequence profiling (25) (Table 3 in Data Set S1) and are a well established model system known to be highly resistant to acidic conditions (30) and can survive in the mammalian stomach (31). Hence, we tested whether acidity and other environmental factors (osmotic and oxidative pressures) regulate E. coli Arg- and Lys-dependent acid resistance systems by quantifying the expression of amino acid decarboxylases, *speA* (32), *adiA* (33), and *cadA* (34) (Table 2; Fig. 4).

**TABLE 2:** Primers used to quantify the level of expression of Arg- and Lys-decarboxylase genes *in vitro*^*a*^

Gene	Primers	PCR fragment size (nt)	Function
*speA*	F_GGTGTACTACGCTCCATG	124	Biosynthetic Arg-decarboxylase involved in putrescine synthesis; pH independent
	R_TAATGTGGCCCAGCTCGT		
*adiA*	F_CTCCATCAAGACACCTGG	140	Degradative Arg-decarboxylase, inducible by low pH in rich media anaerobically
	R_AGGCAGTCAATGGCTTCG		
*cadA*	F_CCATCCGTGAACTTCATC	157	Inducible Lys-decarboxylase, producing cadaverine, a superoxide radical scavenger
	R_ATTTCTTCGCACAGCTCG		

a Expression of Arg- and Lys-decarboxylase genes that are part of E. coli amino acid-dependent acid resistance mechanisms was tested. Total microbiota purified from colorectal cancer and control meta-samples were grown aerobically for 24 h at 37°C. The forward (F) and reverse (R) gene-specific primers for PCR with annealing temperature of 56°C and the sizes of the amplicons are shown. PCR fragments were cloned into the TA-pGEM vector (Promega), and 10 randomly selected clones for each gene after transformation were Sanger sequenced, and BLAST was used to compare against the nucleotide collection database for confirmation of target specificity.

Expression of *speA* (pH-independent Arg-decarboxylase) was positively regulated by E. coli in response to all four growth conditions, irrespective of the health status of the host, except for dl-lactate in the noncancerous samples. This indicates that E. coli of the gut microbiota activate the SpeA resistance pathway in response to acid and non-acid pressures primarily in a health-independent manner. This (i) shows that *speA* transcription is activated in response to salt, oxidative, and inorganic acid pressures, irrespective of health status but to lactate-based acidity in a health-dependent way, and (ii) suggests that gene expression of individual bacteria (such as E. coli) of the gut microbiota is regulated by specific pressures and dependent on the health status of the host. Together, this suggests that the SpeA response represents a broad-spectrum stress protection mechanism of the aerobic gut microbiota.

Expression of *adiA* (biosynthetic Arg-decarboxylase) was enhanced by CRC-derived E. coli regardless of the nature of acidity as opposed to both osmotic and oxidative pressures that downregulated *adiA* expression. In contrast, a mixed picture (downregulation by lactate and salt, upregulation by H_2_O_2_, and no effect by HCl) was observed for control cultures on transcription of *adiA*. This is consistent with the health status of the host being a key mediator of the AdiA-dependent acid stress response mechanism to all but osmotic pressure. Thus, expression of the E. coli AdiA Arg-dependent acid resistance system is differentially regulated by different environmental factors in a host health status-dependent manner.

Expression of *cadA* (pH-inducible Lys-decarboxylase) was positively regulated by either acidic condition regardless of health status, showing that the CadA Lys-dependent acid resistance mechanism operates independently of host health. Osmotic pressure, however, inhibited its expression in only CRC E. coli, while this enhanced expression of the gene in the control culture in the presence of H_2_O_2_, demonstrating that salt and oxidative pressures regulate *cadA* transcription in a manner influenced by host health. Hence, the CadA Lys-dependent acid resistance mechanism is activated in response to acidity in a health-independent manner while playing a role in protection of bacteria of the healthy but not cancerous gut against oxidative stress.

Both Arg- and Lys-dependent acid resistance mechanisms were positively regulated under acid conditions in CRC-derived E. coli regardless of the nature of the acid. However, the Lys-dependent acid resistance mechanism, unlike the Arg-dependent systems, responded in a health-independent manner. It appears that E. coli originating from different microbiomes respond differentially to the same acid stresses. Both amino acid-dependent acid defense systems sensed the oxidative pressure in a health-dependent manner, while the SpeA Arg-dependent subsystem responded, irrespective of the origin of E. coli. Osmotic stress elicited opposite patterns of Arg-dependent system regulation and was not influenced by health status, while the Lys-dependent mechanism displayed health-dependent regulation. *In fine*, this argues that the gut microbiome, at least its aerobic population, responds to the same environmental pressures in a unique fashion depending upon their native gut environment, be it CRC-affected or healthy.

### The CRC-associated microbiome expresses a plethora of virulence and colonization factors.

**FIG 5:**
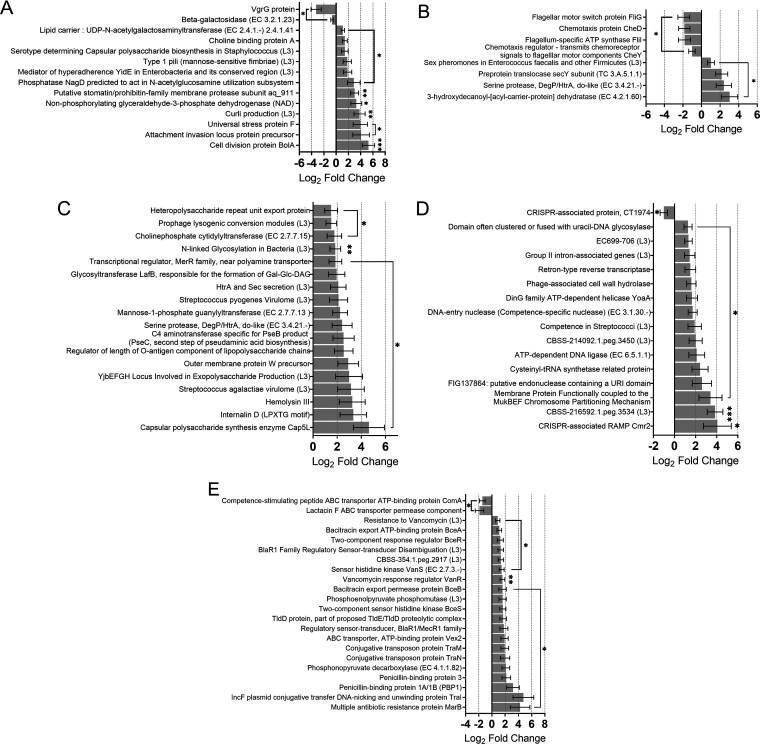
The microbiome in colorectal cancer colonize the host and form biofilms, exchange DNA, and overexpress numerous virulence determinants. (A) Transcription of genes that are important for colonization, flagellin and pilin modifications, and the formation/remodeling of the cell wall (96) was elevated in the CRC microbiome. Higher transcription of *BolA* and the curli production subsystem (which play roles in biofilm formation) and lower transcription of the possible hypoxanthine oxidase *XdhD* and the bifunctional PLP-dependent enzyme with β-cystathionase and maltose regulon repressor activities (which facilitate biofilm disassembly) suggest increased biofilm formation in the CRC-associated microbiome. (B) Quorum sensing (QS) and motility were regulated in CRC. Gram-negative QS-associated genes were overrepresented in CRC, and expression of the *secY* gene, translocase, and *DegP*/*HtrA* serine proteases was higher in CRC. Gram-positive QS mechanisms were, however, attenuated in cancer. Transcription of several chemotaxis and flagellar production/function genes (*CheY*, *FliI*, *FliG*, and *CheD*) was reduced in the CRC niche. (C) The CRC microbiome activate expression of virulence factors. Production of capsular polysaccharide synthesis enzyme Cap5L; heteropolysaccharide repeat unit export protein, *Irp2*, which encodes the iron acquisition yersiniabactin synthesis enzyme (Fig. 3B); hemolysin III; and the LPXTG-containing motif internalin D was increased. Expression of *R*-alcohol forming, (*R*)- and (*S*)-acetoin-specific 2,3-butanediol dehydrogenase (Fig. 3A), which reduces acetoin to 2,3-butanediol, was enhanced in CRC, suggesting a potentially high supply of acetoin, promoting a pro-cancerous phenotype of the CRC-specific microbiota. (D) The CRC gut microbiota are prone to the exchange of genetic information, protective against pervasive bacteriophages and repair errors in their genome. Transcription of a DNA-entry nuclease (a competence-specific nuclease) was increased in CRC. Expression of the CRISPR-associated RAMP *Cmr2* gene, a part of the type III system, and retron-type reverse transcriptase was amplified. However, transcription of the CRISPR-associated protein CT1974, a member of the CRISPR subtype I-E of E. coli (97), was decreased. There was increase in transcription of genes for helicase YoaA (involved in the repair of replication forks), domain clustered with uracil-DNA glycosylase and FIG137864:putative endonuclease domain (involved in releasing damaged pyrimidines from double-stranded DNA [dsDNA]). Higher expression of cysteinyl-tRNA synthetase-related protein in CRC suggests that the RecA-mediated recombinational repair mechanism and hence the SOS response were increased under cancerous conditions. (E) Antibiotic resistance activities of the microbiome are positively regulated in CRC. Increased transcription of the two-component regulatory system VanR/VanS (98), which senses either the presence of extracellular vancomycin and/or cell wall disruption by, e.g., bacitracin, was observed. *Vex2*, encoding an ATP transporter that is important for a vancomycin-tolerant phenotype, was overexpressed. The CRC gut microbiota showed an enhanced expression of *MarB*, a periplasmic protein that may indirectly repress the expression of *MarA*, a trigger of bacterial response to different toxic compounds, including antibiotics (99). β-Lactam resistance of the CRC microbiome appears to be significantly enhanced, as it is seen via greater activity of the BlaR1 family regulatory sensor-transducer disambiguation subsystem. The expression of *BlaR1*/*MecR1* family genes (100) that sense β-lactams and activate expression of β-lactamase PC1/*blaZ* and penicillin-binding proteins 1A/1B and 3 (poorly acylated by β-lactam antibiotics) that confer resistance to the antibiotic was elevated. Activity of the subsystem, phosphoenolpyruvate phosphomutase, and expression of the phosphonopyruvate decarboxylase gene, involved in biosynthesis of fosfomycin, were increased in CRC. Lactacin F ABC transporter permease component, a bacteriocin, was transcribed less in CRC. Horizontal gene transfer facilitated through expression of *ComA*, a member of bacteriocin-associated ATP-binding transporter family, was repressed. However, higher conjugative activity was likely a feature of the microbiome through enhanced transcription of *TraM* and *TraN* genes, as well as the *TraI* gene, encoding *IncF* plasmid conjugative transfer DNA-nicking and unwinding protein. This would enhance genome plasticity and confer more adaptive traits to the microbiota in CRC. *, *P ≤ *0.05; **, *P ≤ *0.01; ***, *P ≤ *0.001.

Taxonomic analysis of this microbiome revealed elevated activity of the oral cavity for *Enterobacteriaceae*, ESKAPE (*Enterococcus faecium, Staphylococcus aureus, Klebsiella pneumoniae, Acinetobacter baumannii, Pseudomonas aeruginosa*, and *Enterobacter* spp.), and other clinically relevant pathogenic species (25); the same community displayed enhanced activity of numerous specific virulence determinants. The CRC microbiota transcribed exopolysaccharide, heteropolysaccharide, and capsular polysaccharide biosynthesis genes more readily (Fig. 5A and C). This suggests that Gram-positive microbes in CRC can colonize the mucosal surface and evade opsonophagocytosis more effectively (35). Furthermore, expression of lipoteichoic acid polymer-forming genes (36), which is anchored to the bacterial membrane, was also promoted. Improved adhesion properties of the microbiota in CRC were evidenced by enhanced activity of type 1 pili and the adherence of *Enterobacteria* subsystems and a number of adhesion-related genes (Fig. 5A). Interestingly, expression of the *VgrG* gene, a component of the type VI secretion system (37), and *YdjG*, a hypothetical oxidoreductase that is required for E. coli colonization (38), was downregulated. This suggests that adhesion/colonization of some pathogenic *Proteobacteria*, such as Pseudomonas, Escherichia, Klebsiella, Burkholderia, and Acinetobacter, does not involve a phage-like secretion mechanism.

We observed enhanced activity of salmochelin-mediated iron acquisition subsystem (Fig. 3B). Salmochelin has been shown to promote both pathogenic E. coli colonization and biofilm formation *in vivo* (39). Increased production of lipopolysaccharide by Gram-negative bacteria in CRC was evident through an increase of pseudaminic acid biosynthesis gene expression (Fig. 5C), which helps microorganisms evade the host immune system (40). Consistent with both enhanced adhesive properties, biofilm formation-associated gene transcription is also promoted. Upregulation of genes involved in the production of curli (Fig. 5A), amyloid fibers that form the extracellular biofilm matrix, is a signature of the CRC-associated microbiota. Our findings suggest that sporulation activities of microbes in CRC were diminished, while early-stage germination seemed to be increased (Text S1; Fig. S5).

The bacterial ability to perform curli-mediated adherence is inversely coordinated with their motility (41) required for movement and adhesion to the mucosa or epithelium (42). Motility- and chemotaxis-related gene expression of the microbiota in CRC was repressed (Fig. 5B). These data argue that colonized and clustered microorganisms are a potential signature of CRC. A key microbial gene, β-galactosidase, which is involved in the degradation of mucus, was downregulated in CRC (Fig. 5A), consistent with the notion that during CRC the microbiota has already colonized the host epithelium and, to a lesser extent, the mucus.

One of many mechanisms that allow microbiome to adapt to environmental changes (43) is the horizontal transfer (HT) of genetic information. HT facilitates the creation of a diverse and fluctuating array of genetic combinations often enforced by selective pressures. Genes required for conjugation, which requires cell-to-cell interaction (44), in Enterococcus and other *Firmicutes* were upregulated (Fig. 5E). HTs in Gram-positive bacteria (competence in Streptococcus and sex pheromones in Enterococcus faecalis and other *Firmicutes*) (Fig. 5B) and DNA repair (CBSS-214092.1.peg.3450 and EC699-706) were all enhanced in CRC (Fig. 5D). Two antiviral defense mechanisms, CRISPR-Cas (the adaptive microbial immune system, CBSS-216592.1.peg.3534) and group II intron-associated genes (preventing phage propagation through the microbial population at the expense of infected microbes, abortive infection) (45), were upregulated in CRC (Fig. 5D). At the transcriptional level, CRISPR type III system was upregulated, while the E. coli CRISPR subtype I-E was downregulated. We found that DNA repair gene expression was augmented in CRC, including ATP-dependent DNA ligase, also crucial for DNA replication and recombination.

Antimicrobial resistance has been a major health-related concern for decades, and subjecting microbial communities to antibiotic pressure plays a major role in the development and spread of these determinants (46, 47). Surprisingly, we found that the gut microbiota of the CRC cohort (who were not subjected to antibiotic treatment in the 2 months preceding sample collection) displayed a multidrug-resistant phenotype (Fig. 5E) via induced expression of *MarB* (48). We also observed enhanced expression of genes conferring resistance to vancomycin and β-lactams. These data demonstrate that the CRC gut can promote expression of antibiotic resistance determinants; this may be due to the enhanced activity of microbes carrying antibiotic resistance, including ESKAPE and *Enterobacteriaceae* species (25).

Furthermore, the CRC microbiota showed a significantly higher transcription of bacitracin transport genes, cyclic peptide antibiotics that disrupt Gram-positive cell wall synthesis. Enhanced gene expression for production of microcin B17, a peptide toxin that causes microbial double-stranded DNA (dsDNA) breaks (49), and fosfomycin, which interferes with cell wall biosynthesis, was also a CRC signature. Interestingly, fosfomycin acts against methicillin- and vancomycin-resistant *Enterobacteriaceae* pathogens with increased susceptibility to the antibiotic in an acidic environment (50), a feature of the malignant gut. The higher production of microbiota-derived antimicrobials suggests increased competition between microorganisms in CRC.

## DISCUSSION

For the first time, we analyzed the active microbiota, via the metatranscriptome, of the CRC gut and found functional dependency of the microbial community on the health state of the host. We showed inherently different regulated responses of gut microbes to diverse environmental factors, depending on whether bacteria were health- or disease-associated. This work revealed distinct mechanisms by which the microbiota are modulated by and modulate the malignant state of the gut.

The gut microbiota, the “germ organ” of the host, is a unique microbial community as it develops with the host from birth. It is well known that, despite the constant interaction of the microbiota with the colonic mucosa, no general inflammation of the gut is observed day to day (51). The observed core functions across cohorts are in concordance with general housekeeping activities that allow microbes to coexist with the host despite their inflammatory potential. The downregulation of the pyruvate:ferredoxin/flavodoxin core subsystem indicates that the microbiome is conducting less anaerobic respiration as a whole. However, certain pathway activities that occur in the absence of oxygen are still observed and in some cases are even upregulated (see below, TME), indicating local areas of hypoxia in the more oxygen-rich CRC niche.

The stepwise accumulation of sporadic genetic lesions causing CRC has been attributed to the damaging effects of ROS (52). Oxidative stress response constitutes pathways that reduce ROS, such as O_2_^−^ and H_2_O_2_ to protect membranes, proteins, and DNA from damage (53). If the damage exceeds the capacity of host DNA repair mechanisms, genetic mutations may occur. H_2_O_2_-dependent dysregulation of epithelial barrier function would facilitate microbial colonization and invasion promoting inflammation and ROS production. However, our work argues that inflammation-derived ROS appears to be only half of the picture. Several unexpected lines of evidence strongly argue that the gut microbiota is a crucial mediator of ROS levels through their ability to scavenge and reduce ROS. If the capacity of microbiota to control the level of physiological ROS (during “homeostasis”) is reduced, even temporarily over 15 to 30 years, this may facilitate the known accumulation of ROS-induced damage (genetic and epithelial barrier function) and hence onset of CRC (54). We infer that expression of the oxidative stress subsystem is a core housekeeping function of the microbiome. In response to enhanced ROS availability, the microbiota are to offset this by increased ROS reduction to lower ROS to physiological levels. Conversely, overinduced activity of microbial antioxidative mechanisms may lead to diminished ROS levels, also causing gut pathology through compromising epithelial barrier integrity (55). We propose that two modes of ROS-mediated genetic damage can occur: (i) chronic or intermittent inflammation-dependent ROS accumulation due to, e.g., inflammatory bowel disease (IBD) or antibiotic treatment and (ii) inflammation-independent damage, in which compromised ROS-reducing microbiome functions lead to excess or diminished ROS. However, these modes may together form one continuous cyclic pathology; the epithelium can be compromised in an inflammation-independent manner leading to inflammation.

Osmotic pressure, which in part regulates synthesis of ectoine, a nonenzymatic antioxidant (21), appears to be a regulated factor that may be associated with elevated levels of chemical antioxidants in the gut. Interestingly, the major reactive species response in the CRC gut appears to be to RNS, specifically nitric oxide. This is in concordance with high levels of inflammation that occur in the cancerous gut, including elevated levels of microbial colonization, resulting in activation of host iNOS (56).

Iron uptake and transport by the CRC microbiota appear to be enhanced. It was shown that tumor cells accumulate iron while blocking its export (57), a likely cause of the deficiency common to the condition. The potential synergistic effect of elevated microbial and tumoral iron uptake could be a mechanism by which the host becomes deficient. Iron supplementation, therefore, may have adverse effects by feeding tumor growth and pathogen virulence, hence posing a greater risk of infection and further inflammation. Many cancer patients suffer from carnitine deficiency, 75% of which is derived from the diet (58). The observed enhanced catabolism of carnitine by microbiota in CRC may explain, at least in part, this phenomenon. Uncontrolled growth of cancerous colonocytes is underpinned by dysregulation and reprogramming of gene expression, including translation (59). Humans rely on dietary scavenging and the gut microbiota for their supply of Q (queuosine, 7-deazaguanosine), necessary for tRNA (Q_34_tRNA) to ensure translation fidelity (60). Elevated microbial transport of Q suggests they are assimilating queuosine at a higher level and potentially depleting the host of this vital molecule, thus decreasing the accuracy of host protein synthesis.

Our data argue for local regions of hypoxia and O2 saturation, consistent with the known architecture and metabolism of the TME (61) and biofilms (28). These conditions facilitate sequential colonization of oxygen-respiring microbes in proximity to tumor blood vessels (and local areas of inflammation) and facultative and obligate anaerobes further from the O2 supply (61). The TME toward the blood vessels is less acidic (CO2, H+, and lactate are vented into the bloodstream) and enriched with oxygen. Further from the vasculature, the TME becomes more hypoxic and acidic. Under this O2 gradient, cancer cells become more glycolytic and release lactate and protons into the surrounding lumen, forcing anaerobes to modify their membrane structure with unsaturated fatty acids to decrease H+ permeability, a trait we observed being adopted by the microbiota (Text S1). This suggests close interaction of microbial subpopulations coinhabiting specific niches that cannot support the growth of anaerobes and aerobes simultaneously. However, under oxygen-rich conditions, anaerobes can still thrive in CRC via formation of biofilms, with obligate anaerobes being the primary colonizers, forming the inner biofilm layers, which become hypoxic following colonization of other bacteria (62).

The present analysis of microbial RNA-seq data revealed evidence that the CRC gut environment, compared to its healthy counterpart, is more acidic. This can be due, in part, to the altered metabolism of cancerous colonocytes, which excessively produce lactate even in the presence of oxygen, namely, aerobic glycolysis or the Warburg effect (63). This would acidify the gut tumor microenvironment, hence affecting metabolism of the microbiota and potentially adapting them to this pressure. Here, we showed that the CRC gut microbiota enhanced expression of glutamate decarboxylase (GAD) of the GAD-dependent acid defense mechanism (64). This defense is similar to other microbial acid resistance mechanisms, such as Arg- and Lys-decarboxylase systems, which produce basic compounds and consume protons, hence increasing cytoplasmic pH (65). E. coli strains, whether pathogenic or not, are abundant constituents of the gut microbiota, are remarkably well equipped with acid resistance mechanisms, and can cause different diseases, including infections born from contaminated acidic food (34).

The Lys-dependent mechanism appears to be a universal acid defense system that protects gut bacteria against acid, irrespective of the health status of the host or nature of the acid. CadA decarboxylates Lys to cadaverine, a superoxide antioxidant, which is exported from cells in exchange for extracellular lysine and thus alkalinizing the cytoplasm by consuming a proton. However, the CadA system in the non-acid-adapted microbiotas is either unresponsive to or repressed by high salinity while playing an adaptive role in response to oxidative stress in a health-dependent manner. This suggests that the Lys-dependent resistance mechanism may provide the gut microbiota with additional nonenzymatic protection against ROS in response to high acidity and hydrogen peroxide availability, while activation by the latter is health status-dependent. In contrast, both Arg-dependent systems responded differently to both acid pressures and in a health-dependent manner. Both subsystems appear to sense low pH (H^+^ and lactate) only if they originated from the CRC microbiome, confirming that the Arg-dependent systems are important for maintaining pH homeostasis of aerobic microbiota in CRC. It has been shown that expression of *adiA* is triggered only under anaerobic growth at low pH (66). Our data clearly showed that *adiA* is expressed under aerobic conditions by E. coli of gut microbiota in a pH-independent manner regardless of the health status of origin. One possible explanation could be that growth of a complex mixture of aerobic microbes reduces the level of oxygen in the medium, resulting in derepression of the E. coli AdiA system. Additionally, low pH is not required for derepression of transcription of Arg and Lys acid-dependent systems in aerobic conditions. A potential cross-communication of microbiota and/or acidification of the medium due to CO_2_ production (e.g., by the activity of pH-independent SpeA) may be sufficient to maintain a constitutive level of expression of these amino acid defense mechanisms in an aerobic environment *in vitro*. Expression of *adiA* appears to be also regulated by salt and oxidative pressures. Interestingly, upregulation was observed only in microbiota derived from the control samples and in response to H_2_O_2_. These data further suggest that microbiota adapt to the environment of the gut and can exhibit these inherited properties later by regulating their patterns of gene expression as part of survival strategies, perhaps through transcriptional memory. *speA* appears to be a broad-spectrum stress defense mechanism, at least in E. coli under aerobic conditions. However, the health status of the host also affects the ability of the SpeA-mediated mechanism to maintain pH homeostasis of the cell, supporting the view that the microbiota of the CRC and noncancerous gut are fundamentally different. Acids, salt, and reactive oxygen species trigger *in vitro* amino acid-dependent acid resistance mechanisms in a health-dependent manner, suggesting that these factors are features of the CRC human gut that in turn direct microbial acid tolerance, consistent with our metatranscriptome data.

The microbiota evolved numerous adaptive mechanisms by which they can exchange and expand their genetic information. One of these mechanisms includes bacteriophage infection through lysogeny facilitated by their cohabitation (67). We also observed that the microbiota in the cancerous gut promote HT, a prominent feature of biofilms (28). Furthermore, activities of two major antiviral defense mechanisms can trigger abortive infection, namely, retrons and the broad range (naive) CRISPR-Cas type III system (68) coinciding with phage lysogeny/prophage expression in the microbiome in CRC. It is also known that bacteriophages facilitate HT (69), and our findings suggest this may be the case to a greater degree in the cancerous gut. Interestingly, the CRC-associated gut microbiota downregulate CRISPR-Cas type I while enhancing activity of CRISPR-Cas type III. Type I responds to previously acquired invasive mobile genetic elements (iMGEs) and can target HT (70). However, the type I CRISPR system is known to fail in recognition of new and mutated sequences, allowing accumulation of invader escapers like prophages (68). Greater uptake of extracellular DNA by the gut microbiota and higher activity of a wide range of bacteriophages in CRC would be consistent with enhanced activity of a type III CRISPR-Cas system.

CRC-associated microbiota display a higher degree of resistance to two clinically relevant antibiotics: the glycopeptide vancomycin and the β-lactam penicillin. This implies that the gut environment may be a previously unrecognized pressure selecting for certain resistance mechanisms. Vancomycin resistance can be triggered by nonantibiotic changes occurring on outer membranes (71). These resistances further reduce therapeutic options, particularly for methicillin-resistant Staphylococcus aureus (MRSA), coagulase-negative staphylococci, and other Gram-positive infections in penicillin-allergic individuals (72). Our data argue that bacterial competition is enhanced in CRC through production of antimicrobials, bacitracin (73), microcin (49), and fosfomycin (74) and appears to be a primary feature of their cohabitation. Higher activity of the multiple antibiotic resistance phenotype and some efflux transporters is consistent with the CRC gut being more toxic to the microbiota.

The present work characterizes for the first time the functions and adaptative responses of the gut microbiota in colorectal cancer. As ROS are a primary trigger of CRC development, the ability of the microbiota to modulate ROS levels in the gut poses some important questions. For example, what are the environmental signals that can regulate microbial antioxidative activities: e.g., diet, antibiotics, toxins, or other pressures? Another important task is to characterize the specific pressure(s) that promote the enhanced antibiotic resistance phenotype displayed during disease. This is critical for patients who require surgery to prevent postoperative infection. Understanding and subsequently manipulating such adaptive mechanisms that the microbiota uses to compete for nutrients, exchange genetic material, and control prevalence and activity of other gut species can be a useful tool in developing bacteria-based therapy.

This work provides direct links between specific adaptive responses of the gut microbiome in the colorectal cancer gut via metatranscriptomics. Our findings reveal important insights into the protective role of gut microbiome against developing cancer and its adaptive responses to the tumor environment. A striking example is the high background level of microbial-mediated ROS reduction activities in both CRC and healthy gut microbiomes, an apparent “core housekeeping” role of the gut community, protecting colonocytes against ROS-induced DNA damage and promoting epithelial integrity. Our data show that the CRC and control gut microbiota adapt through inherently different mechanisms to environmental pressures of the gut *in vitro*. This shows that the health status of the microbiota host controls microbial adaptation to specific stresses, laying the foundation for investigation into effective strategies for microbial manipulation. Depletion of the gut for beneficial metabolites in combination with enhanced genetic exchange, virulence, host colonization, and antibiotic and acid resistance in colorectal cancer make the microbiome more pathogenic and less protective.

## MATERIALS AND METHODS

### Sample collection.

Fecal samples from CRC patients and volunteers collected under the auspices of the Famished study at the University Hospital Coventry and Warwickshire National Health Service (NHS) Trust (UHCW) (United Kingdom ethics certificate 09/H1211/38). Fecal samples from 10 CRC patients (requiring emergency surgery) and 10 randomized non-CRC volunteers were collected at UHCW. The samples were immediately frozen in liquid nitrogen upon collection and stored at –80°C. Patient metadata were also collected at UHCW (Table S1).

10.1128/msphere.00627-22.7 TABLE S1 Clinical metadata of the participants collected by University Hospital Coventry and Warwickshire NHS Trust. Download Table S1, XLSX file, 0.01 MB. Copyright © 2023 Lamaudière et al. 2023 Lamaudière et al. https://creativecommons.org/licenses/by/4.0/ This content is distributed under the terms of the Creative Commons Attribution 4.0 International license.

### RNA and DNA isolation and sequencing.

The RNeasy PowerMicrobiome kit (Qiagen) for total RNA extraction was used following manufacturer protocol; 300 mg of each fecal sample was used. Purified total RNA was stored at –80°C. Total RNA quality and concentration was analyzed using the Agilent Technologies 2100 Bioanalyzer capillary gel electrophoresis system. RNA-seq was carried out by Vertis Biotechnologie AG, Germany, including depletion of rRNA, preparation of cDNA and Illumina NextSeq 500 sequencing (2 × 150 bp paired-end sequencing to produce 2 × 420 M reads). The cDNA inserts were flanked with the following adapter sequences, TruSeq_Sense_primer, i5 Barcode 5′-AATGATACGGCGACCACCGAG ATCTACAC-NNNNNNNN-ACACTCTTTCCCTACACGACGCTCTTCCGATCT-3′ and TruSeq_Antisense_primer, i7 Index 5′-CAAGCAGA AGACGGCATACGAGAT-NNNNNNNN-GTGACTGGAGTTCAGACGTGTGCTCTTCCGATCT-3′. DNA was extracted from the 300-mg fecal samples using DNeasy PowerSoil Pro kit (Qiagen) following manufacturer protocol. Blank extractions (300 μL of water) were carried out to assess the quality of the DNA and RNA extraction kits, and this did not yield any detectable nucleic acids. Total DNA was stored at –80°C. 16S rRNA gene V3-V4 regions were sequenced by Novogene Co., Ltd. on Illumina (NovaSeq 6000 PE150) paired-end platform (100,000 tags of raw data per sample) to generate 250-bp paired-end raw reads (Raw PE), merged, and pretreated to obtain clean tags. Clean tags were removed to obtain the effective tags. Operational taxonomic units (OTUs) were obtained by clustering with >97% identity on the effective tags of all samples; taxonomic annotation was made for the representative sequence of each OTU to obtain the corresponding taxa information and taxa-based abundance distribution.

### Metatranscriptome data processing and analysis.

Raw reads were processed following the steps of the SAMSA2 (75) (version 2.2.0) pipeline. First, read pairs were trimmed to remove low quality bases using Trimmomatic (76) (version 0.36), and then overlapping read pairs were merged into single sequences using PEAR (77) (version 0.9.11). These sequences were “ribodepleted” *in silico* using SortMeRNA (78) (version 2.1) to identify and remove sequences representing rRNA. These ribodepleted sequences were translated and assigned to functional classes of the SEED subsystems hierarchical database (79) using DIAMOND (80, 81) (version 0.8.38) to align reads against a database of 7,939,855 protein sequences. Alternatively, these ribodepleted sequences were mapped to “taxonomic expansion” sequences of the RefSeq database (82). Sequences assigned to genes of each functional class were aggregated to give raw abundance count data for each class. These counts were used to determine statistically significant differential abundance of functional classes (83) between CRC and control conditions using DESeq2 (84) (version 1.26.0) with *P* values adjusted via the Benjamini-Hochberg false discovery rate method (85) (false discovery rate [FDR] < 0.1). To profile contributions of bacterial species to biochemical pathways, HUMAnN3 (86) (version 3.0.1) was run for each set of *in silico* ribo-depleted sequences from control and CRC groups. To maximize the number of results returned, the DNA and translated DNA coverage thresholds (defaults 50% and 90%) were removed. Output files were merged into a single table using the “humann_join_tables” function. Count data were normalized to control for differing sequencing depths using the “humann_renorm_table” function to normalize all levels by the community total, including any unmapped, unintegrated and ungrouped features and representing counts as copies per million (CPM).

### Expression of E. coli amino acid resistance genes under different growth conditions *in vitro*.

Fecal bacterial isolation, DNA and RNA extraction was conducted as described previously (25). Levels of expression of E. coli *adiA*, *cadA*, and *speA* genes in the CRC and control microbiota cultures (from pooled total fecal bacteria, for each cohort) in response to acids, osmotic, and oxidative pressure were measured by quantitative reverse transcription (qRT)-PCR, with annealing temperature of 56°C for all reactions, as described (25). Mann-Whitney U tests for qRT-PCR (*P < *0.05) were conducted to establish statistically significant differences in gene expression between the CRC and control groups. qRT-PCR was conducted using gene specific primers (Table 2), and 16S rRNA gene primers were used for normalization as described (25). Primer specificity was confirmed by Sanger sequencing by Eurofins after cloning PCR fragments into a TA pGEM-T Eazy cloning vector, Promega as described (25). Additionally, the SYBR Green iTaq (Bio-Rad) qRT-PCR system was tested with the 16S rRNA gene primers for contamination (water as the template) and DNA contamination of the RNA samples (proportionally to the amount of cDNA used for amplification diluted RNA samples were used as the templates for PCR). No amplification was observed for all control samples.

### Ethics approval and consent to participate.

This study was approved by University Hospital Coventry and Warwickshire NHS Trust, United Kingdom ethics certificate No. 09/H1211/38. All volunteers provided informed consent prior to participation and for the publication of any research results.

### Data availability.

All data were submitted to the European Nucleotide Archive (ENA) under project accession No. PRJEB53891. Statistical outputs of data analyses are available as supplemental tables.
