# Supplementary figures and images for: The Colorectal Cancer Microbiota Alter Their Transcriptome To Adapt to the Acidity, Reactive Oxygen Species, and Metabolite Availability of Gut Microenvironments
Source: mSphere. 2023 Feb 27;8(2):e00627-22. doi: 10.1128/msphere.00627-22 (PMC10117117; doi:10.1128/msphere.00627-22)

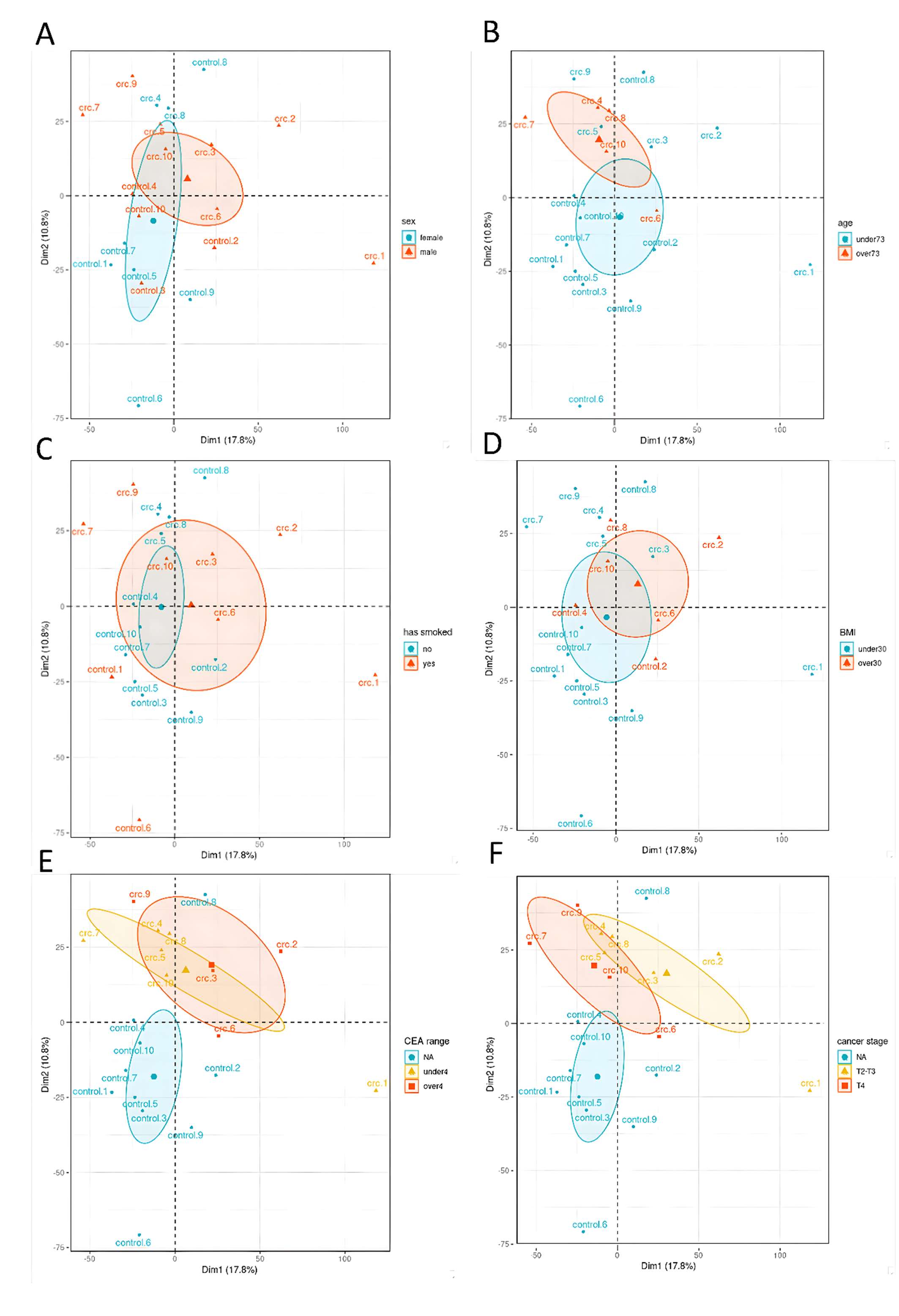

Supplement: FIG S1 [file msphere.00627-22-s0001.tif]

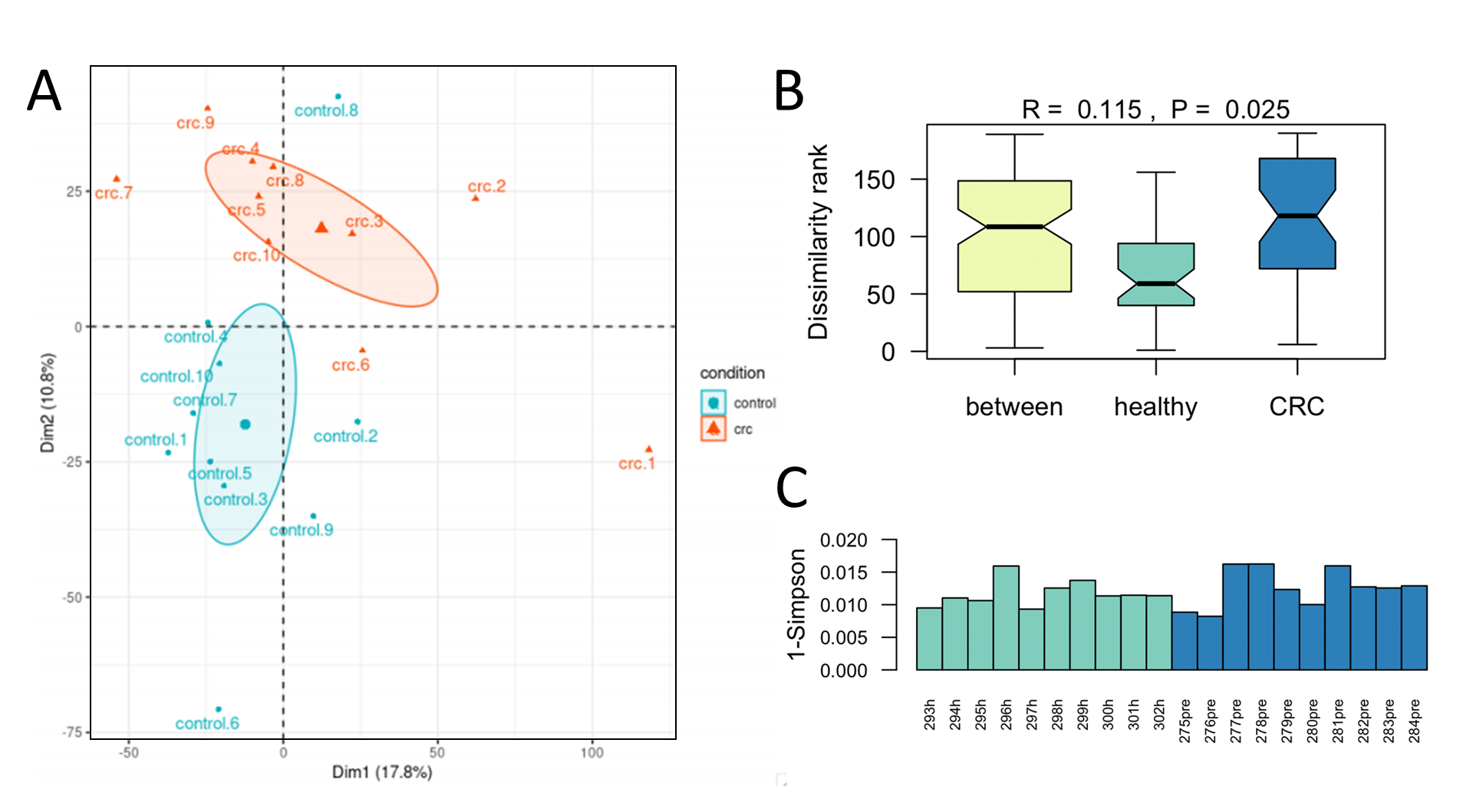

Supplement: FIG S2 [file msphere.00627-22-s0002.tif]

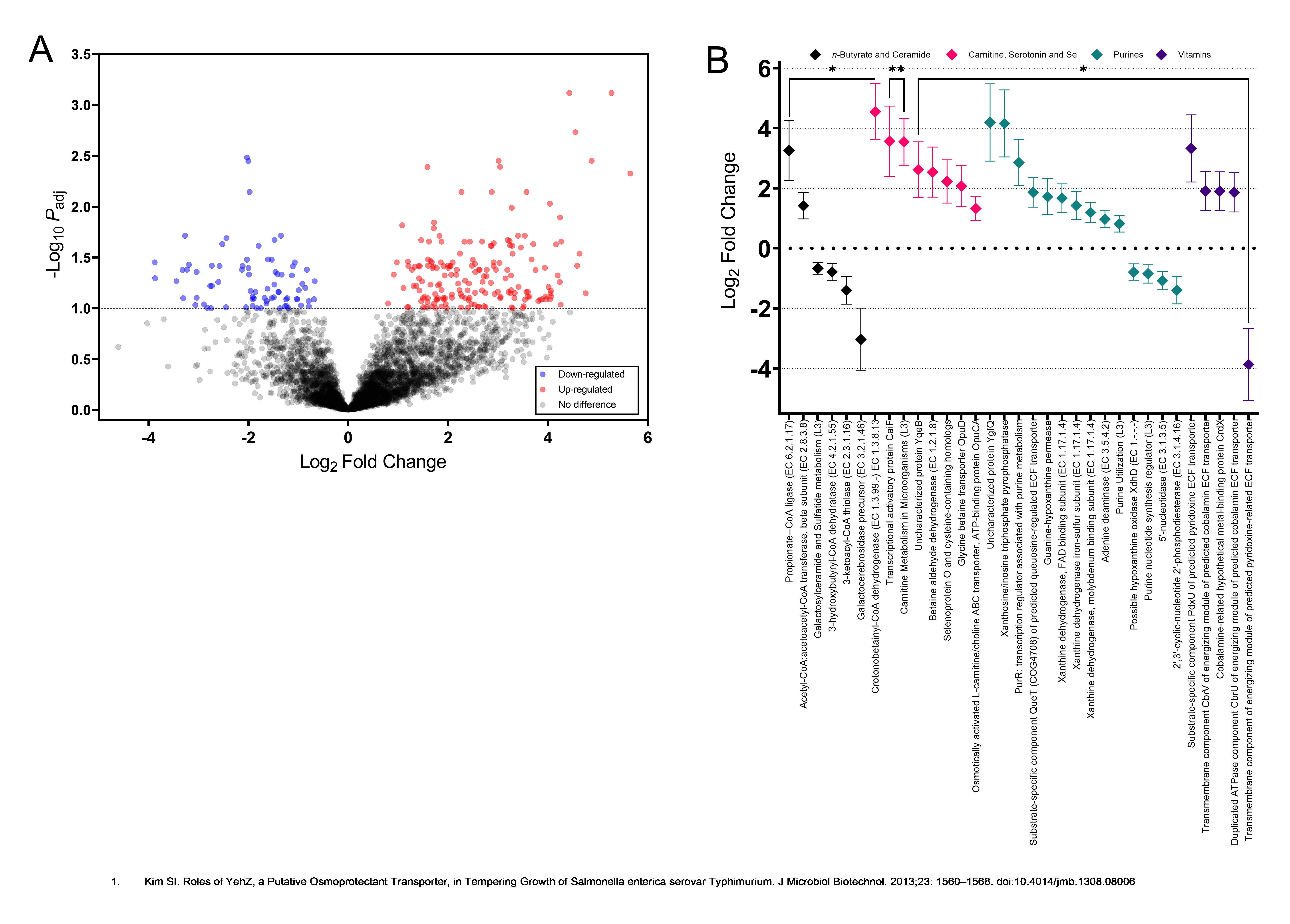

Supplement: FIG S3 [file msphere.00627-22-s0003.tif]

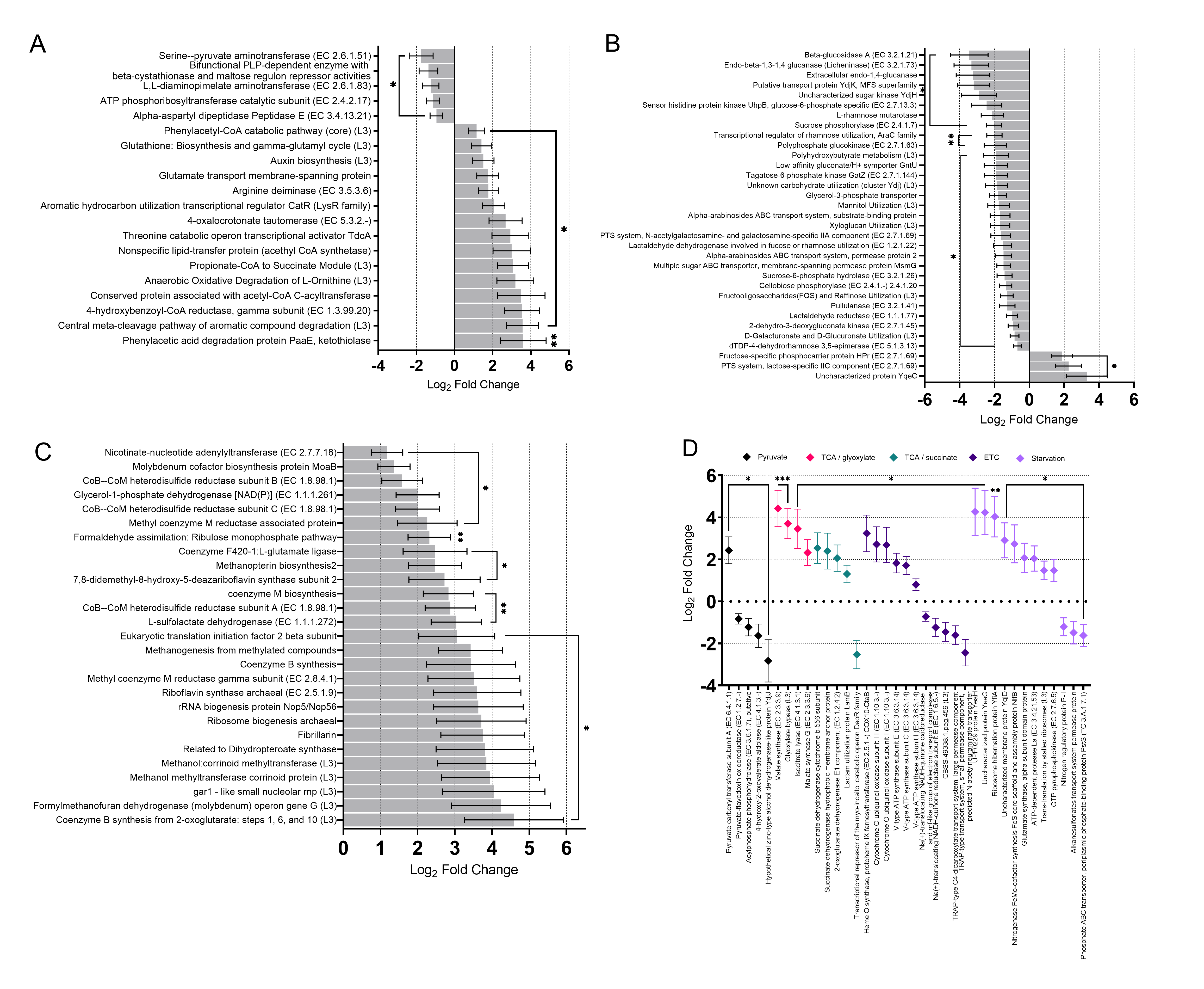

Supplement: FIG S4 [file msphere.00627-22-s0006.tif]

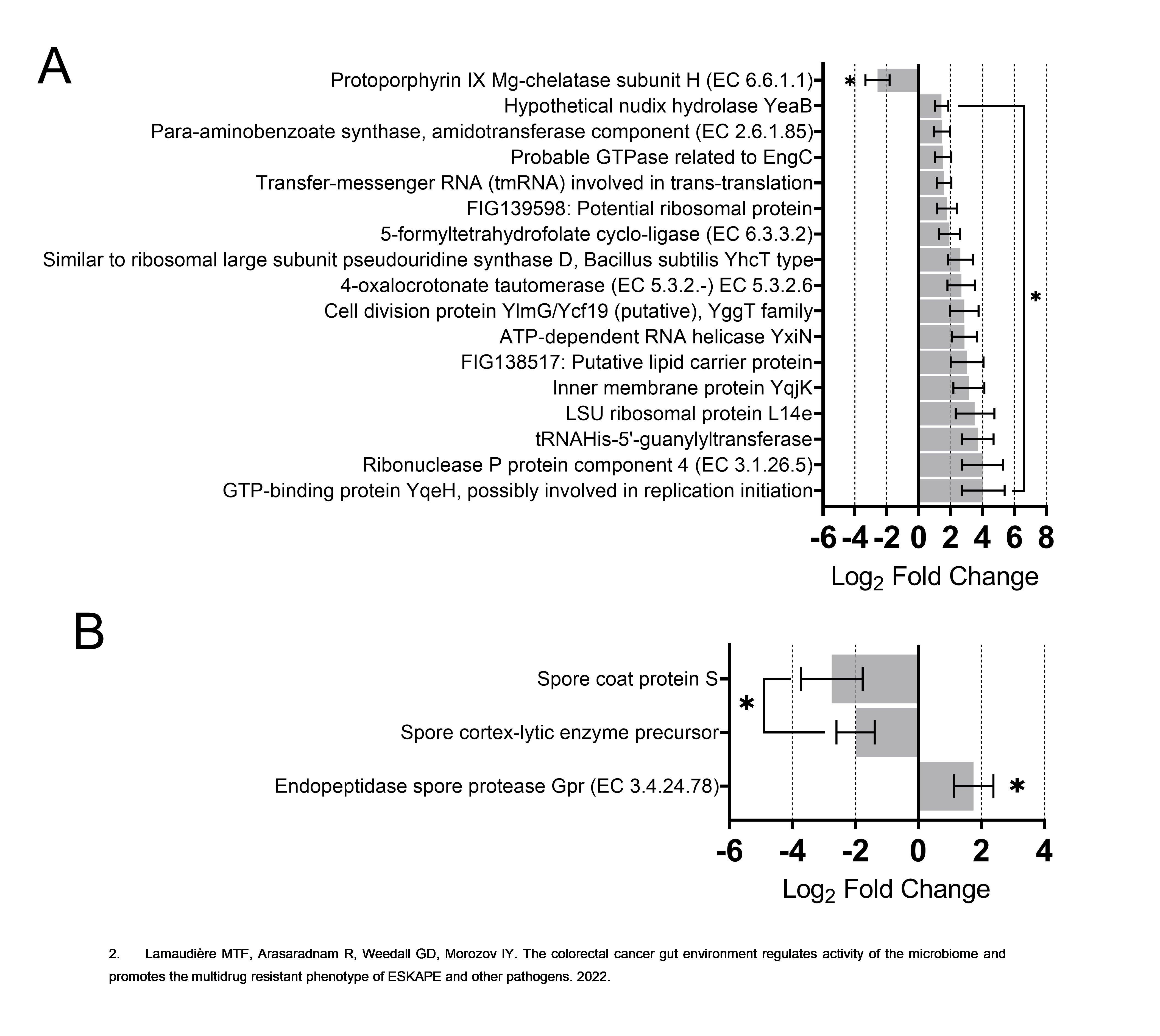

Supplement: FIG S5 [file msphere.00627-22-s0007.tif]
