# Supplementary material for: The Colorectal Cancer Microbiota Alter Their Transcriptome To Adapt to the Acidity, Reactive Oxygen Species, and Metabolite Availability of Gut Microenvironments
Source: mSphere. 2023 Feb 27;8(2):e00627-22. doi: 10.1128/msphere.00627-22 (PMC10117117; doi:10.1128/msphere.00627-22)
Supplement: TEXT S1 [file msphere.00627-22-s0005.docx]

**The Colorectal Cancer Microbiota Alter Their Transcriptome To Adapt To the Acidity, Reactive Oxygen Species and Metabolite Availability of Gut Microenvironments**

**Matthew T. F. Lamaudière^a^, Ramesh Arasaradnam^b, c, d^, Gareth D. Weedall^f#^ and Igor Y. Morozov^a#^**

^a^Centre for Sports, Exercise and Life Sciences, Coventry University, Coventry, UK

^b^Divison of Biomedical Sciences, Warwick Medical School, University of Warwick, Warwick, UK

^c^ Department of Gastroenterology, University Hospitals of Coventry and Warwickshire, NHS trust, Coventry, UK

^d^University of Leicester, Leicester, UK

^f^School of Biological and Environmental Sciences, Liverpool John Moors University, Liverpool, UK

Running Head: Microbial metatranscriptome and colorectal cancer

#Address correspondence to Igor Y. Morozov [ab6069@coventry.ac.uk](mailto:ab6069@coventry.ac.uk)

Or Matthew Lamaudière ([lamaudi2@uni.coventry.ac.uk](mailto:lamaudi2@uni.coventry.ac.uk))

**Supplementary Results and Discussion**

**The CRC-associated microbiota deplete the supply of *n*-butyrate to the mitochondria**

It is well established that the gut microbiota produces *n*-butyrate, a major SCFA, by fermentation of non-digestible dietary carbohydrates. This microbial-derived metabolite is the primary energy source of colonocytes which catabolize it in the mitochondria via β-oxidation, hence helping to maintain the strict anaerobic environment of the gut and promote growth of beneficial anaerobic bacteria. Unlike healthy epithelial cells, cancerous colonocytes do not utilize *n*-butyrate for their growth, instead preferring other carbon and energy sources (1). *n*-Butyrate is produced by two major bacterial metabolic pathways, via either phosphorylation of butyryl-CoA to *n*-butyrate by butyrate kinase or the butyryl-CoA:acetate CoA-transferase (acetyl-CoA:acetoacetyl-CoA transferase) route (2) The activity of butyrate kinase was comparable across cohorts (data not shown) while transcription of the acetyl-CoA:acetoacetyl-CoA transferase (β-subunit) gene was significantly increased in CRC (Fig. S4), suggesting the synthesis of *n*-butyrate from butyryl-CoA and acetate is enhanced. However, synthesis of crotonyl-CoA (from 3-hydroxybutyryl-CoA), the substrate for acetyl-CoA:acetoacetyl-CoA transferase, was significantly reduced due to repressed expression of the crotonase gene. Despite the enhanced activity of acetyl-CoA:acetoacetyl-CoA transferase, the potential depletion of crotonyl-CoA and hence, butyryl-CoA may switch the substrate specificity from butyrate production towards interconversion of acetoacetate and acetoacetyl-CoA for acetone and/or acetyl-CoA, the latter appears to be in sufficient supply. This is consistent with our recent observation that activity of 22 major butyrate producing bacteria in CRC is significantly reduced (3). The observed metabolic activities of the gut microbiota in CRC, with respect to the supply of beneficial micronutrients, indicates significant overall reductions. Furthermore, we found a high level of carnitine utilization (Fig. SB) by microbiota in CRC, a further strong indication of restricted supply of the major mitochondrial fatty acid carrier which is crucial for β-oxidation, hence limiting the ability of colonocytes to utilize *n*-butyrate in CRC.

**Nucleic acids are metabolized to a greater degree by the CRC microbiome**

Purine utilization by the CRC gut microbiota appears to be more pronounced (Fig. S3B). This includes enhanced expression of genes which are involved in (i) uptake of guanine and hypoxanthine bases, (ii) purine degradation, (iii) cleaning up of the nucleotide pool and (iv) regulation of purine metabolism (4). This suggests the CRC microbiome salvages xanthosine/inosine nucleosides as an energy-saving alternative and to maintain the integrity of the genome (e.g. during replication) and may use these purines as C- and N- sources to compensate for diminished carbohydrate metabolism. Consistent with the activation of the *PurR* operon, microbial purine nucleotide synthesis seemed to be repressed during CRC (purine nucleotide synthesis regulator subsystem). However, conversion of adenine to guanine nucleotides and production of adenosines via hydrolysis of AT(D/M)P and mRNA degradation, were downregulated in CRC, suggesting that microbes do not use an adenine/adenosine salvage pathway as a C- and energy source in the cancerous gut.

**The CRC microbiota adjust their respiratory pathways to utilize amino acids and aromatic compounds, alter H^+^ transport in the low pH milieu and divert resources towards biosynthesis**

Microbes have evolved several mechanisms to utilize a wide variety of carbon and nitrogen sources, often cross-feeding on the products of metabolism of others. Our analysis shows dynamic metabolism of amino acids and carbohydrates. Anaerobic oxidative degradation of L-ornithine, producing acetate, alanine and tumour-promoting ammonia (important for bacterial colonization) (5) appears to be a signature of the CRC microbiota (Fig. S4A). However, anaerobic metabolism of complex carbohydrates such as D-galacturonate and D-glucuronate derived from pectin and other heteropolysaccharide was repressed. Upregulation of archaeal methanogenesis genes, the anaerobic endpoint of fermentation, the acetoin, butanediol metabolism module suggests that some members of the microbial community grow under hypoxia and use fermentation for energy production to a greater degree. We found that expression of several genes that support aerobic metabolism under oxygen-limited settings, in other words, associated with growth under microaerobic conditions, was enhanced in CRC.

A recent large meta-analysis of CRC gut metagenomes has revealed a significant enrichment in pathways of amino acid degradation alongside a depletion of genes for carbohydrate utilization (6). We found that the activity of these pathways reflected the reported altered metagenome abundances (Fig. S4A and S4B). Interestingly, enhanced degradation of some amino acids may serve another adaptive purpose, namely protection of microorganisms against low environmental pH. An acidic environment can favour the growth of archaea, and we saw a host of pathways involved in the biosynthesis of archaeal ribosomes, coenzyme M and B and methanopterins which were more active in the CRC gut (Fig. S4C). This data coupled with methanogenesis from methylated compounds and formaldehyde assimilation subsystems and archaeal vitamin B_2_ synthesis upregulation, all point to a cancerous gut with a flourishing archaeome. Methanogenesis, the anaerobic endpoint of fermentation, the process of reducing CO_2_ into CH_4_, methane, in the presence of H_2_ for the purpose of energy production is a prominent CRC signature. We also observed enhanced utilization of aromatic compounds as the primary C and N sources, shown through CRC-dependent expression of e.g. 4-oxalocrotonate tautomerase involved in the central meta-cleavage pathway for aromatic compound degradation, a subsystem with augmented activity alongside the phenylacetyl-CoA catabolic pathway (Fig. S4A). Degradation of branched amino acids to propionate-CoA and L-lysine catabolism was also a distinct signature of the CRC microbiota as seen through upregulation of both the propionate-CoA to succinate module (Fig. S4A) and lysine degradation (Fig. 3A) subsystems. Transcription of glutathione metabolizing genes, involved in amino acid transport as part of the γ-glutamyl cycle (7) were amplified. Notably, enhanced amino acid uptake by CRC-associated microbiota coincided with a high level of acidity. Expression of acid-induced amino acid transport genes and inner membrane transport protein YbaT (Fig. 2D) and glutamate/aspartate uptake were all up-regulated (Fig. 3A). Transcription of genes needed for degradation of Arg, Ser/Thr metabolism and Phe catabolism were also elevated. Trp degradation appeared to be promoted too, as expression of the auxin biosynthesis subsystem and monoamine oxidase gene was greater. Contrarily, activity of His, Lys and D-amino acid biosynthesis genes and α-aspartyl dipeptidase peptidase E was significantly reduced in CRC.

Such high catabolic activity of amino acids in CRC appears to result in starvation of e.g. Ser. Transcription of the serine-pyruvate aminotransferase gene was decreased (Fig. S4A) while expression of the GTP pyrophosphokinase protein which catalyzes formation of pppGpp, the precursor of ppGpp, an alarmone active in response to amino acid starvation (8) was enhanced (Fig. S4D). The observed amino acid starvation in turn may trigger the elevated ribosomal hibernation response observed, through production of membrane protein YqjD and ribosome hibernation protein YfiA.

Interestingly, microorganisms in CRC activated expression of glutamine synthetase, a member of the ubiquitous pathway for ammonium assimilation (9). Downregulation of the structural gene for nitrogen regulatory protein P-II, that regulates the catalytic activity and transcription of glutamine synthetase was observed in CRC (Fig. S4D). This suggests that glutamine synthetase-dependent N-assimilation from ammonium is a preferred nitrogen source in CRC to support high cell growth under nitrogen-limited conditions. By contrast, the CRC gut appeared enriched with phosphate as expression of *PstS* (a phosphate ABC transporter) was repressed, its transcription is known to be negatively regulated by the availability of phosphate.

Genes encoding multiple sugar ABC transporters, as well as lactaldehyde reductase and dehydrogenase responsible for L-fucose and L-rhamnose degradation were expressed to a lesser extent in CRC. We found that expression of 24 genes, activities of which are required for carbohydrate transport and metabolism, was negatively correlated with CRC (Fig. S4B). Interestingly however, genes involved in fructose metabolism exhibit enhanced transcription, required for nucleic acid biosynthesis, consistent with increased biosynthetic metabolism.

A dramatic change in microbiome metabolism from utilization of carbohydrates to amino acid catabolism supposes two important physiological characteristics of the cancerous gut. Firstly, a high demand for glucose and other simple sugars by proliferating tumour cells to sustain uncontrolled growth is consistent with a lower availability of simple carbohydrates for microbes. Secondly, it is very unlikely that the diet of patients would be significantly altered comparing with the control group (patients were scheduled for emergency surgery, therefore could not alter long-term dietary habits). Therefore, supply of carbohydrates should remain. However, during cancer, pathogens such as Enterobacteriaceae and ESKAPE pathogens that do not metabolize fibre, are more active in CRC (3) and can increase their colonization due to an expansion of the microaerobic niche, which may in-part lead to the decreased activity of saccharolytic *Bacteroides* and some *Clostridium* (10) Genomes of saccharolytic species encode several hydrolases which can degrade complex carbohydrates (11) hence supplying simple sugars to other microbes (cross-feeding). Increased activity of pathogenic bacteria at the expense of carbohydrate hydrolysing species may also contribute to a deficiency of simple sugar metabolism and force microbiota to utilize amino acids, nucleotides and aromatic compounds as alternative carbon sources. Moreover, extracellular DNA can also be utilized as a nutrient source to offset the lack of simple carbohydrates in the cancerous gut (12). We cannot rule out that inflammation, nutrient depletion and/or biofilm formation in the cancerous gut (13) also add a further factor that induces DNA uptake.

Both aerobic and anaerobic respiration entails glycolysis, pyruvate metabolism, tricarboxylic acid production and oxidative phosphorylation using either oxygen as the terminal electron acceptor or less energetic molecules, e.g. nitrate, sulfate, fumarate. The TCA cycle is fed with a supply of pyruvate from various sources, the glycolytic Embden-Meyerhof-Parnas (EMP) pathway chief among them. However, glucose availability appears limited during CRC, reflected in attenuated microbial glycolysis (Fig. S4D). The gluconeogenic pyruvate carboxylase can be activated by the allosteric regulator acetyl-CoA and high pyruvate concentrations. This initiates production of glucose from non-carbohydrates, such as lactate and amino acids and converts pyruvate to oxaloacetate instead of acetyl-CoA and displayed a marked rise in expression, again suggesting lack of glucose and potentially glutamate (the allosteric inhibitor of the enzyme) availability (14). Interestingly, production of glyceraldehyde-3-phosphate (GADP) in both ED and EMP glycolytic pathways was augmented (Fig. 5A), perhaps facilitating host mucin adhesion, if it is cell wall-associated (15) by the microbiota rather than playing a crucial role in energy generation. Expression of pyruvate producing oxo-acid lyase, 4-hydroxy-2-oxovalerate aldolase, was down-regulated in CRC, hence the production of acetaldehyde, an inhibitor of gluconeogenesis, was suppressed alongside supply of pyruvate. Further evidence of enhanced gluconeogenesis, and thus higher activity of biosynthetic pathways, this was supported by upregulation of the propionate-CoA to succinate module. Propionate can feed gluconeogenesis though the TCA cycle after conversion to succinate-CoA followed by oxidation to glucose via pyruvate and oxaloacetate. β-Oxidation of odd carbon fatty acids and the catabolism of Iso, Val, Met and Thr amino acids are major sources of propionate-CoA, supporting our findings of increased amino acids catabolism by the CRC microbiota. The microbiota favoured oxidation of the limited glucose for anabolic purposes through the pentose phosphate pathway as seen through elevated transcription of *YqeC*, implicated in production of the second NAD(P)H cofactor within the pathway, necessary for reductive biosynthetic reactions in fatty acid, aromatic amino and nucleic acid production. This data overall shows that the CRC microbiota may deploy gluconeogenesis, amino acid catabolism and specific nucleotides to offset the deficiency of simple sugar availability.

Transcription of a key β-oxidation of fatty acids enzyme 3-ketoacyl-CoA thiolase was downregulated by the CRC microbiota (Fig. S3B), suggesting the catabolism of amino acids and not β-oxidation of fatty acids was a primary source of acetyl/propionate-CoA. The two decarboxylating steps (isocitrate → 2-oxoglutarate → succinyl-CoA) of the TCA cycle can be circumvented via the glyoxylate bypass, this shunt is preferred upon a metabolic switch from energy to biomass producing activity and was more active in CRC (Fig. S4D). The preferred glyoxylate bypass in accordance with the anabolic pentose phosphate pathway argues that the microbiota have high biosynthetic demands during colorectal cancer, potentially facilitated by their access to colonocytes triggering microbial proliferation.

Succinate, the major substrate for oxidative phosphorylation during respiration can be produced not only through the TCA cycle and glyoxylate bypass, but also through the γ-aminobutyrate (GABA) shunt, converting 2-oxoglutarate to succinate, bypassing succinyl-CoA and NADH production in the process. This TCA cycle re-entry point was potentially utilized more readily by the gut microbiota in cancer, via lactam utilization protein, LamB (Fig. S4D). Once more, the propionate-CoA to succinate module demonstrates elevated activity, supplying succinate. However, dietary carboxylic sugar, *myo*-inositol, can also be catabolized to e.g. propionate, acetate, CO_2_, acetaldehyde and succinate by commensal microbes (16) which also displayed upregulation through diminished expression of the transcriptional repressor of the *myo*-inositol catabolic operon *deoR*. These data argues that there is an abundance of succinate, supplied more actively by four routes, GABA and glyoxylate shunts, the propionate-CoA to succinate module and *myo*-inositol catabolism, which the CRC microbiota can utilize to provide electrons to the electron transport chain in respiration during cancer and satiate their biosynthetic requirements.

The observed upregulation of the glyoxylate bypass would result in less NADH, one of two electron donors required for Complex I to catalyze electron transfer to ubiquinone. This, coupled with Na(+)-translocating NADH-quinone reductase subunit E and the FMN-producing pathway under-transcription (Fig. S4D) by the cancerous microbiome, suggests Complex I of the electron transport chain has a diminished role in cellular respiration during cancer. However, transcription of genes encoding for enzymes which carry out electron transfer from Complex II through to Complex V was higher in CRC. Complex II, where multiple component-encoding genes display elevated expression, alongside ample succinate availability suggests Complex II is the entry point for the generally more active electron transport chain in CRC.

The cancer-associated microbiota was also more active with respect to translation, cell division/replication, cleansing the nucleotide pool, folding of bacterial chromosomes, and daughter cell separation (Fig. S5A). Together with more pronounced gluconeogenesis and biosynthetic metabolism in CRC, an enhanced level of microbial growth in the cancerous gut is also supported by our findings. This includes enhanced biosynthesis of NAD(+) cofactor, folate (vitamin B_9_) and nucleotides, membrane phospholipids and their carriers and peptidoglycan as well as methylation (SAM MTase) in CRC. Overexpression of some serine protease and hydrolase genes which are implicated in the prevention of misfolded proteins and potentially toxic nucleoside diphosphate derivative accumulation in fast growing cells was observed.

**Quorum sensing activities of Gram-positive and Gram-negative bacteria are diminished and elevated, respectively, and less spore formation occurs during CRC**

We observed significant changes in transcription of a small group of genes required for sporulation and germination (Fig. S5B). The CRC gut environment may suppress sporulation and support germination of sporulated bacteria under certain conditions. This may be a consequence of the local environment and signals therein. Quorum sensing (QS) activity which responds to growth conditions (17) was affected. Two lines of evidence argue that the Gram-negative QS machinery was upregulated in CRC. Primarily, enhanced transcription of SAM MTase (Fig. 3A) which suggests a greater supply of SAM in CRC and is indirect evidence that excess SAM in the cancerous gut can also stimulate production of homoserine lactone by acyl-homoserine lactone (AHL) pheromone synthases. Secondarily, expression of 3-hydroxydecanoyl-[*a*cyl-*c*arrier-*p*rotein, ACP] dehydratase implicated in C_10_-AHL production was also augmented. The CRC-associated microbiota further displayed QS-mediated regulation of gene expression, such as enhanced production of proteases, e.g. HtrA and Sec secretion subsystem (Fig. 5B and 5C). Contrarily, the peptide-based QS system of Gram-positive bacteria was repressed in CRC.

**A range of virulence determinants are overexpressed by a more inflammatory yet evasive microbial population in the CRC gut**

The CRC-associated microbiota upregulated production of a number of virulence factors. Activities of the *S. pyogenes* and *S. agalactiae* virulomes, prophage lysogenic conversion modules (expression of foreign genes can enhance the pathogenic capability of bacteria) (18) auxin biosynthesis (a virulence factor which is implicated in suppression of plant defence) (Fig. S4A) were more pronounced in CRC. Microorganisms enhanced expression of genes whose products synthesise and transport inflammation-promoting intermediates. Greater microbial transport of polyamines, via upregulation of spermidine putrescine ABC transporter permease component PotB (Fig. 3C) and transcriptional regulator, MerR family, near polyamine transporter (Fig. 2E), suggests an amplified abundance of inflammatory polyamines (19). This has been shown to facilitate both biofilm formation in the colonic mucosa and cancer cell growth, invasion and metastasis (20).

Overproduction of O-antigen of LPS in Gram-negative bacteria, via upregulation of mannose-1-phosphate guanylyltransferase and a regulator of O-antigen component length of LPS chains, is consistent with the enhanced pro-inflammatory properties of the CRC microbiota. Accelerated degradation of phenylacetic acid, an anti-inflammatory catabolite of phenylalanine, through phenylacetic acid degradation protein PaaE (Fig. S4A) may further increase the pro-inflammatory environment of the gut. Acetoin, a potential pro-inflammatory intermediate of bacterial fermentation, the availability of which in the CRC gut appeared to be higher (Fig. 3A), can induce IL-8, IL-6 (pro-inflammatory cytokines interleukins 8 and 6) and TNF-α production, causing significant loss of epithelial barrier function (21). Acetoin can also promote colonization/biofilm formation of environmental microbes in the lungs, including *Pseudomonas aeruginosa* (22) (due to acetoin cross-feeding by other bacteria). It can be reduced to 2,3-butanediol under oxygen limited conditions. Gram-negative facultative anaerobes, such as *Klebsiella*, *Pseudomonas* and *Enterobacter* activities of which have been significantly enhanced in CRC (3) under anaerobic conditions can divert carbon flux from production of organic acids to neutral products, such as 2,3-butanediol. This suggests that acidification of the gut during CRC has a pleotropic effect on microbial metabolism, from enhanced amino acid catabolism to supporting a pro-cancerous gut environment. By contrast, butanediol fermentation in Gram-positive bacteria was attenuated, transcription of *YdjL* (Fig. 3A), putative oxidoreductase, a *Bacillus* *subtilis* 2,3-butanediol dehydrogenase homologue (23) was repressed. This suggests that major 2,3-butanediol-producing Gram-positive bacteria like *Bacillus* spp. and potentially *Streptococcus* spp. are more tolerant to low pH and still can produce mixed acids from pyruvate during cancer. This data *in fine* strongly suggests that microbiota of the gut community in CRC interacts with host cells and expresses diverse virulence factors to facilitate their colonisation.

Elevated production of microbial virulence factors may be a consequence of a higher level of microbial access to and colonization of the epithelium. The observed overexpression of hemolysin III (Fig. 5C), a virulence determinant, may help microorganisms, e.g. members of *Bacteroides fragilis* group, to colonize the CRC gut (24). Hemolysins are known to be central in developing systemic infections by opportunistic pathogens, weakening the immune system and gaining advantages in a competitive niche. Critically, hemolysins lyse and kill host cells, and hence provide a supply of nutrients e.g. nucleic acids. This is consistent with our finding that the cell death rate in the CRC gut is elevated (Fig. 3C). Induced production of capsular polysaccharides and EPS results in more hydrophilic properties of the cell wall surface which was shown to be less susceptible to phagocytosis by neutrophils (25) facilitating microbial immune evasion. Furthermore, biosynthesis of pseudaminic acid (26) (Fig. 5C) supports the assertion that an array of adaptative approaches is employed by the microbiota in CRC to evade host immune cells.

**References**

1. Donohoe DR, Collins LB, Wali A, Bigler R, Sun W, Bultman SJ. 2012. The Warburg Effect Dictates the Mechanism of Butyrate-Mediated Histone Acetylation and Cell Proliferation. Mol Cell 48:612–626.

2. Liu H, Wang J, He T, Becker S, Zhang G, Li D, Ma X. 2018. Butyrate: A Double-Edged Sword for Health? Advances in Nutrition 9:21–29.

3. Lamaudière MTF, Arasaradnam R, Weedall GD, Morozov IY. 2022. The colorectal cancer gut environment regulates activity of the microbiome and promotes the multidrug resistant phenotype of ESKAPE and other pathogens.

4. Cho B-K, Federowicz SA, Embree M, Park Y-S, Kim D, Palsson BØ. 2011. The PurR regulon in Escherichia coli K-12 MG1655. Nucleic Acids Res 39:6456–6464.

5. Pruss KM, Enam F, Battaglioli E, DeFeo M, Diaz OR, Higginbottom SK, Fischer CR, Hryckowian AJ, van Treuren W, Dodd D, Kashyap P, Sonnenburg JL. 2022. Oxidative ornithine metabolism supports non-inflammatory C. difficile colonization. Nat Metab 4:19–28.

6. Wirbel J, Pyl PT, Kartal E, Zych K, Kashani A, Milanese A, Fleck JS, Voigt AY, Palleja A, Ponnudurai R, Sunagawa S, Coelho LP, Schrotz-King P, Vogtmann E, Habermann N, Niméus E, Thomas AM, Manghi P, Gandini S, Serrano D, Mizutani S, Shiroma H, Shiba S, Shibata T, Yachida S, Yamada T, Waldron L, Naccarati A, Segata N, Sinha R, Ulrich CM, Brenner H, Arumugam M, Bork P, Zeller G. 2019. Meta-analysis of fecal metagenomes reveals global microbial signatures that are specific for colorectal cancer. Nat Med 25:679–689.

7. Orlowski M, Meister A. 1970. The γ-Glutamyl Cycle: A Possible Transport System for Amino Acids. Proceedings of the National Academy of Sciences 67:1248–1255.

8. Boehm A, Steiner S, Zaehringer F, Casanova A, Hamburger F, Ritz D, Keck W, Ackermann M, Schirmer T, Jenal U. 2009. Second messenger signalling governs Escherichia coli biofilm induction upon ribosomal stress. Mol Microbiol 72:1500–1516.

9. Arcondéguy T, Jack R, Merrick M. 2001. P II Signal Transduction Proteins, Pivotal Players in Microbial Nitrogen Control. Microbiology and Molecular Biology Reviews 65:80–105.

10. Litvak Y, Byndloss MX, Bäumler AJ. 2018. Colonocyte metabolism shapes the gut microbiota. Science (1979) 362:eaat9076.

11. Kaoutari A el, Armougom F, Gordon JI, Raoult D, Henrissat B. 2013. The abundance and variety of carbohydrate-active enzymes in the human gut microbiota. Nat Rev Microbiol 11:497–504.

12. Mell JC, Redfield RJ. 2014. Natural Competence and the Evolution of DNA Uptake Specificity. J Bacteriol 196:1471–1483.

13. Lasry A, Zinger A, Ben-Neriah Y. 2016. Inflammatory networks underlying colorectal cancer. Nat Immunol. Nature Publishing Group https://doi.org/10.1038/ni.3384.

14. Bhagavan NV, Ha C-E. 2015. Carbohydrate Metabolism II, p. 205–225. *In* Essentials of Medical Biochemistry. Elsevier.

15. Deng Z, Dai T, Zhang W, Zhu J, Luo X, Fu D, Liu J, Wang H. 2020. Glyceraldehyde-3-Phosphate Dehydrogenase Increases the Adhesion of Lactobacillus reuteri to Host Mucin to Enhance Probiotic Effects. Int J Mol Sci 21:9756.

16. Bui TPN, Mannerås-Holm L, Puschmann R, Wu H, Troise AD, Nijsse B, Boeren S, Bäckhed F, Fiedler D, deVos WM. 2021. Conversion of dietary inositol into propionate and acetate by commensal Anaerostipes associates with host health. Nat Commun 12:4798.

17. Chhabra SR, Philipp B, Eberl L, Givskov M, Williams P, Cámara M. 2004. Extracellular Communication in Bacteria, p. 279–315. *In* .

18. Little JW. 2014. Lysogeny, Prophage Induction, and Lysogenic Conversion, p. 37–54. *In* Phages. ASM Press, Washington, DC, USA.

19. Babbar N, Gerner EW. 2010. Targeting Polyamines and Inflammation for Cancer Prevention, p. 49–64. *In* .

20. Johnson CH, Dejea CM, Edler D, Hoang LT, Santidrian AF, Felding BH, Ivanisevic J, Cho K, Wick EC, Hechenbleikner EM, Uritboonthai W, Goetz L, Casero RA, Pardoll DM, White JR, Patti GJ, Sears CL, Siuzdak G. 2015. Metabolism Links Bacterial Biofilms and Colon Carcinogenesis. Cell Metab 21:891–897.

21. Al-Sadi R, Ye D, Boivin M, Guo S, Hashimi M, Ereifej L, Ma TY. 2014. Interleukin-6 Modulation of Intestinal Epithelial Tight Junction Permeability Is Mediated by JNK Pathway Activation of Claudin-2 Gene. PLoS One 9:e85345.

22. Nguyen M, Sharma A, Wu W, Gomi R, Sung B, Hospodsky D, Angenent LT, Worgall S. 2016. The fermentation product 2,3-butanediol alters P. aeruginosa clearance, cytokine response and the lung microbiome. ISME J 10:2978–2983.

23. Nicholson WL. 2008. The Bacillus subtilis ydjL (bdhA) Gene Encodes Acetoin Reductase/2,3-Butanediol Dehydrogenase. Appl Environ Microbiol 74:6832–6838.

24. Zafar H, Saier MH. 2021. Gut Bacteroides species in health and disease. Gut Microbes 13.

25. Cerning J. 1990. Exocellular polysaccharides produced by lactic acid bacteria. FEMS Microbiol Lett 87:113–130.

26. Chidwick HS, Fascione MA. 2020. Mechanistic and structural studies into the biosynthesis of the bacterial sugar pseudaminic acid (Pse5Ac7Ac). Org Biomol Chem 18:799–809.
